# The Osteoblastic Microenvironment Determines the Fate of Breast Cancer Cells Disseminated in the Bone Marrow

**DOI:** 10.1002/advs.202509980

**Published:** 2026-02-06

**Authors:** Hong‐Li Wang, Rui Zhang, Xiao‐Min Yue, Jie Zhou, Yu‐Fan Huang, Rong Meng, Yu‐Li Wang, Xiao‐Qing Li

**Affiliations:** ^1^ Department of Biochemistry and Molecular Biology Tianjin's Clinical Research Center for Cancer Key Laboratory of Breast Cancer Prevention and Therapy of the Ministry of Education Tianjin Medical University Cancer Institute and Hospital, National Clinical Research Center for Cancer Tianjin China; ^2^ Department of Clinical Laboratory Tianjin Medical University General Hospital Tianjin China

**Keywords:** bone metastasis, breast cancer, dormancy, osteoblastic microenvironment

## Abstract

Bone is the most common destination of metastatic breast cancer cells. Upon dissemination to the bone, cancer cells may either colonize aggressively or enter a quiescent state, depending on interactions with the bone microenvironment. This study revealed how the osteoblastic microenvironment determines the fate of cancer cells disseminated in the bone marrow. Cancer cells remain quiescent as disseminated tumor cells (DTCs) or as micrometastases within an inactive osteoblastic microenvironment (homeostasis) but colonize the bone in an active, nonmineralized osteoblastic (osteogenic) microenvironment. In a highly mineralized osteoblastic microenvironment, basal‐like tumor cells remain quiescent, whereas luminal‐like cancer cells survive and invade the bone. These findings provide a comprehensive explanation for the divergent outcomes of disseminated cancer cells in the bone, focusing on whether they colonize, reside in quiescence, or reactivate from dormancy. Moreover, in a supportive osteogenic microenvironment, both cancer cells and well‐differentiated osteoblasts were demonstrated to activate osteoclasts, leading to osteolytic lesions. Cellular (osteoblasts) and matrix (bone matrix) components exhibited distinct roles in bone colonization. Furthermore, the therapeutic potential of disrupting integrin‐mediated interactions between tumor cells and the bone matrix was evaluated in animal experiments to prevent the reactivation of quiescent tumor cells and their colonization of the bone.

## Graphical abstract

This study revealed how the osteoblastic microenvironment determines the fate of cancer cells disseminated in bone, with a focus on whether they colonize, reside in quiescence, or reactivate from dormancy. Targeting integrin signaling may offer promising strategies for preventing quiescent cancer cells reactivation and bone colonization.

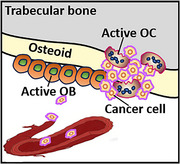

## 1 Introduction

Bone is the most frequent destination of metastatic breast cancer cells [1]. The bone microenvironment plays a crucial role in determining the fate of cancer cells that have spread to the bone marrow. A supportive microenvironment facilitates cancer cell survival and proliferation within the bone, contributing to the development of early bone metastases. Conversely, an unsuitable bone microenvironment can lead to cancer cell quiescence until changes in the microenvironment favor the awakening of dormant cancer cells, resulting in the formation of late bone metastases. Quiescent tumor cells may exist either as individual disseminated tumor cells (DTCs) in a long‐term dormant state with G0/G1 arrest or as small clusters of micrometastases in a counterbalance between proliferation and apoptosis, resulting in no net change in size [2, 3, 4]. The progression from single DTCs to bone micrometastases represents a critical early stage of bone colonization prior to osteolysis [5, 6, 7]. Importantly, quiescence represents an unexploited therapeutic window to intervene in and eliminate DTCs and micrometastases, as conventional chemotherapy and radiotherapy approaches have limited efficacy against these slow‐cycling cell populations [8, 9]. Therefore, investigating the mechanisms governing cancer cell colonization, dormancy maintenance, and reawakening in the bone marrow may provide potential therapeutic targets.

Runt‐related transcription factor 2 (RUNX2), a key transcription factor for the osteogenic lineage, is enriched in bone‐tropic breast cancer cells [10] and has a high potential for promoting bone metastasis [11, 12]. In our previous study, breast cancer cells with high RUNX2 expression were identified as bone‐tropic seeds capable of being recruited by osteoblasts and of colonizing bone in an active osteoblastic (osteogenic) premetastatic niche (PMN) [10, 13]. However, these RUNX2‐high cancer cells do not exhibit survival or colonization advantages in the bones of unmodified mice [13]. Given that the osteoblast lineage comprises a spectrum of cellular states, ranging from inactive osteoblast precursors and bone‐lining cells to mature osteoblasts and terminally differentiated osteocytes [14], we wondered whether the differentiation status of the osteoblastic microenvironment determines the fate of cancer cells in the bone marrow.

In adult women, the differentiation status of the osteoblastic microenvironment and bone mineral density are systemically regulated by hormones, cytokines, and the intake of vitamin D and calcium (Ca^2+^). Estrogen serves as one of the primary protectors of bone mass, maintaining bone mineral density by stimulating osteoblastic bone formation and suppressing osteoclast‐mediated bone resorption [15]. Parathyroid hormone (PTH) is essential for maintaining calcium and bone homeostasis. While PTH stimulates both bone formation and bone resorption, short‐term exposure to low doses of PTH promotes osteoblast differentiation and increases bone formation [16]. PTH(1‐34), the N‐terminal fragment of human PTH, has been approved for the treatment of osteoporosis because of its ability to promote bone formation by converting osteoblast progenitors and inactive lining cells into mature osteoblasts [17, 18]. Calcium is fundamental for bone metabolism, primarily by providing the mineral essential for bone mineralization. Dietary calcium deficiency impairs the mineralization process, leading to a net accumulation of unmineralized bone matrix (osteoid) [19]. Additionally, the long‐term intake of high‐dose glucocorticoids, such as dexamethasone (DEX), suppresses the maturation and function of committed osteoblasts [20].

In this study, we precisely established diverse osteoblastic microenvironments by using varying doses of PTH and estrogen in combination with a low‐calcium diet and dexamethasone. RUNX2‐overexpressing breast cancer cells were used as bone‐tropic seeds to investigate how the differentiation status of the osteoblastic microenvironment affects the fate of cancer cells disseminated in the bone marrow. Specifically, our study provides a comprehensive explanation for the divergent outcomes of disseminated cancer cells in bone, with a focus on whether they colonize bone, enter a quiescent state as DTCs or micrometastases, or reactivate from dormancy. Additionally, we elucidated the distinct roles of the cellular components (osteoblasts) and matrix components (bone matrix) in bone colonization of breast cancer. Furthermore, we evaluated the therapeutic potential of disrupting the interaction between tumor cells and the bone matrix in animal experiments by preventing quiescent tumor cell reactivation and bone colonization.

## 2 Results

### 2.1 RUNX2 Facilitates the Enrichment of Quiescent Basal‐Like Breast Cancer Cells Within the Homeostatic Bone Microenvironment

**FIGURE 1:**
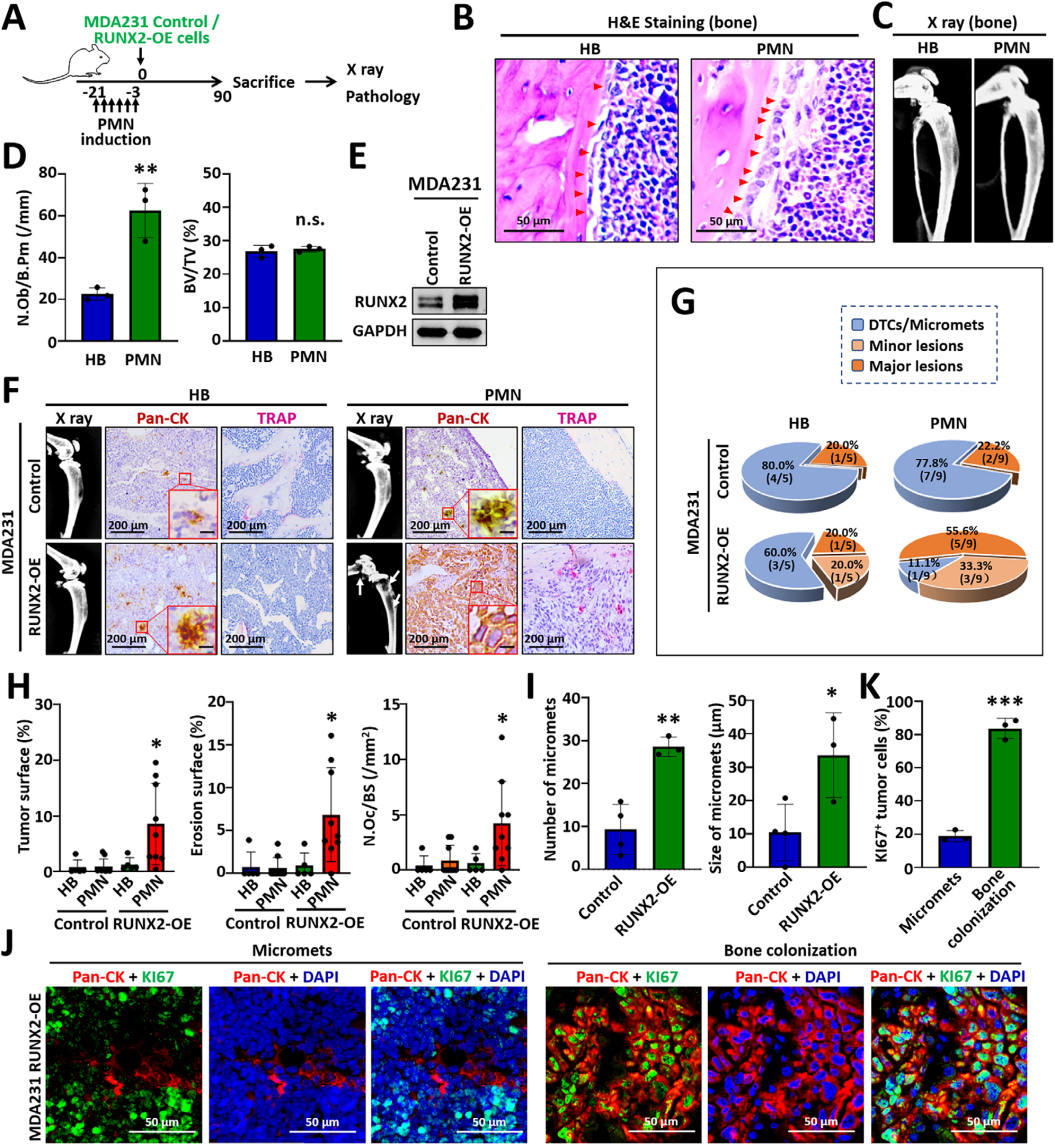
RUNX2 increases the accumulation of MDA231 cells as micrometastases in bone marrow. (A) Experimental schedule for the bone colonization of MDA231‐derived cells in SCID mice. An osteogenic premetastatic niche (PMN) was established by injecting MDA231 CDH11^high^/ITGA5^high^ extracellular vesicles into SCID mice via the tail vein for 3 weeks (2 doses/week). MDA231 RUNX2‐OE cells and control cells were injected into the mice via the left ventricle. (B) H&E staining images demonstrating osteoblasts (indicated by red triangles) in homeostatic bone (HB) and the PMN. (C) X‐ray images showing the bone mass in the HB and the PMN microenvironments. (D) Bar charts quantifying the osteoblast number as the ratio of osteoblast counts to bone perimeter in /mm (N.Ob/B.Pm) and bone mass as the bone volume fraction (BV/TV). (E) Western blot analysis demonstrating increased RUNX2 protein levels in MDA231 RUNX2‐OE cells compared with those in control cells. (F) Representative X‐ray images, pan‐cytokeratin (pan‐CK) immunohistochemical staining images, and TRAP staining images showing osteolytic lesions, tumor cell distribution, and activated osteoclasts, respectively. (G) Pie charts depicting the incidence of DTCs, micrometastases (micromets), and osteolytic lesions formed by control cells and RUNX2‐OE cells within HB and the PMN. The scale bars in the inset images indicate 20 µm. (H) Bar charts quantifying the tumor surface, erosion surface, and the number of TRAP^+^ osteoclasts normalized to the total bone surface (N.Oc/BS in mm^2^). (I) Bar charts illustrating the abundance and size of micrometastases in the bone marrow of mice without detectable bone lesions. (J) Representative KI67 fluorescence immunohistochemical staining images. Pan‐CK was used to label the tumor cells, while DAPI was used to stain the nuclei. (K) Bar chart illustrating the reduced numbers of KI67^+^ tumor cells in micrometastases compared with tumor cells within bone colonization. The data are displayed as the means ± SDs. n.s., not significant; ^*^ *p* <0.05, ^**^ *p* <0.01, ^***^ *p* <0.001 compared with the corresponding controls, as determined by Student's t‐test.

In our previously published study [13], tumor‐derived extracellular vesicles enriched with high levels of both cadherin 11 and integrin α5 (CDH11^high^/ITGA5^high^) were well elucidated to induce an osteogenic PMN characterized by activated osteoblasts and increased production of bone matrix proteins. In the present study, we also observed an increase in osteoblast number and changes in osteoblast morphology in the premetastatic niche, but no change in bone mass (Figure 1A–D; Figure S1A–D). Under homeostatic (inactive) conditions, osteoblasts on the endosteal surface appeared as a single layer of flat cells, whereas in the active PMN, they became enlarged and exhibited a spindle‐shaped, conical, or columnar morphology (Figure 1B, Figure S1B).

In homeostatic bone, both MDA‐MB‐231 (MDA231) cells and 4T1 cells with RUNX2 overexpression presented slight increases in osteolytic colonization capacity compared with their respective control cells, accompanied by a limited extent of osteoclast activation (Figure 1E–H; Figure S1E–H). In mice without osteolytic lesions, control cells were observed surviving as DTCs or small micrometastases in the bone marrow (Figure 1F; Figure S1F). Moreover, both RUNX2‐overexpressing MDA231 and 4T1 cells formed more frequent and larger micrometastases in the bone marrow (Figure 1I; Figure S1I). These micrometastases presented low KI67 expression, indicating limited proliferative capability (Figure 1J,K; Figure S1J,K). Notably, within the osteogenic PMN, RUNX2‐overexpressing cells (RUNX2‐OE) formed significant bone lesions (Figure 1F–H; Figure S1F–H). Overall, these findings suggest that RUNX2 facilitates the enrichment of basal‐like breast cancer cells as quiescent micrometastases within the homeostatic bone microenvironment of adult mice, promoting their progression to overt metastases and osteolytic lesions in a supportive osteogenic microenvironment.

Although both DTCs and micrometastases are indolent, micrometastases are regarded as an intermediate stage in the progression from DTCs to overt bone metastasis [5, 6, 7]. Thus, RUNX2‐overexpressing cancer cells are more likely to become activated and form overt metastases in a supportive bone microenvironment and were therefore utilized in our subsequent study to investigate the influence of the osteoblastic microenvironment on cancer cells.

### 2.2 An Unmineralized Osteogenic Microenvironment Facilitates the Colonization of Basal‐Like RUNX2‐OE Breast Cancer Cells in Bone

**FIGURE 2:**
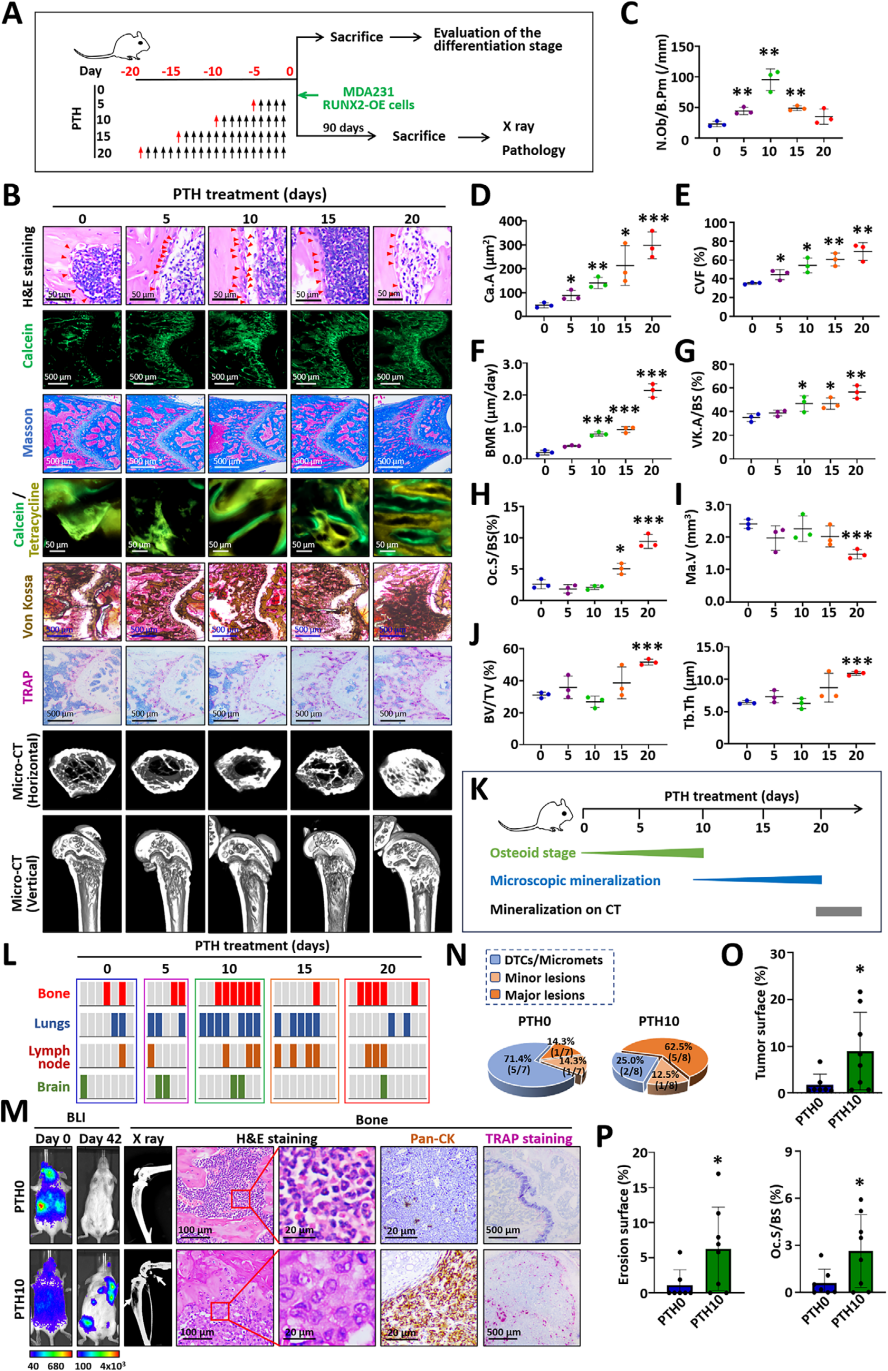
The differentiation status of the osteoblastic microenvironment affects the colonization of MDA231 RUNX2‐OE cells. (A) Experimental diagram illustrating the bone colonization of cancer cells in mice following parathyroid hormone (PTH) treatment. SCID mice received daily intraperitoneal injections of 100 µg/kg PTH for 0, 5, 10, 15, or 20 days, followed by the evaluation of the differentiation of osteoblastic microenvironment or the injection of MDA231 RUNX2‐OE cells via the left ventricle. Tetracycline hydrochloride (30 mg/kg) was administered on days 13 and 14, and calcein (6 mg/kg) was injected on days 3 and 4 prior to sacrifice for evaluation of the osteoblastic microenvironment. (B) Representative H&E staining, calcein fluorescence, Masson's staining, calcein/tetracycline fluorescence space, Von Kossa staining, TRAP staining, and micro‐CT images. The red triangles in the H&E‐stained images indicate osteoblasts. (C) Strip plot qualifying the osteoblast number as N.Ob/B.Pm. (D) Plot of the percentage area of calcein fluorescence (Ca.A) normalized to the bone surface area. (E) Strip plot depicting the collagen volume fraction (CVF) quantified by Masson's staining. (F) Strip plot displaying the bone mineralization rate (BMR) calculated from the calcein/tetracycline fluorescence space. (G) Strip plot illustrating the area of Von Kossa staining (VK.A) normalized to the bone surface area. (H) Plot of the percent TRAP^+^ surface area normalized to the bone surface area (Oc.S/BS). (I) Plot of marrow volume (Ma.V) reflecting cortical bone thickness. (J) The bone volume fraction (BV/TV) and trabecular thickness (Tb.Th) were determined from micro‐CT images. (K) Characterization of the stages of the osteoblastic microenvironment in mice induced by PTH for various durations. (L) Chart showing the colonization of cancer cells in the bone (red bars), lungs (blue bars), lymph nodes (brown bars), and brain (green bars) in mice treated with PTH. (M) Representative bioluminescence (BLI) images demonstrating the systemic distribution of cancer cells in SCID mice on day 0 and the multiorgan colonization observed on day 42. Additional representative X‐ray, H&E, immunohistochemical staining for pan‐CK, and TRAP staining images that indicate bone lesions, tumor cell distribution, and osteoclast activity at the time of sacrifice are shown. (N) Pie charts depicting the incidence of DTCs, micrometastases, and osteolytic lesions in mice treated with PTH for 10 days (PTH10) and in control mice (PTH0). (O) Bar chart showing the tumor surface normalized to the bone surface. (P) Bar charts indicating the erosion surface area and TRAP^+^ surface normalized to the bone surface. The data are presented as the means ± SDs. ^*^ *p* < 0.05, ^**^ *p* < 0.01, and ^***^ *p* < 0.001 compared with the PTH0 group, as determined by Student's t test.

Given that the osteoblast lineage comprises various cells at different stages of differentiation, we investigated whether the differentiation status of the osteoblastic microenvironment affects the colonization of cancer cells. We administered PTH to the mice and established diverse osteoblastic microenvironments by varying the treatment duration (Figure 2A). The differentiation status of these osteoblastic microenvironments was then evaluated on the basis of osteoblast activity, osteoid accumulation, calcium deposits, and mineralized bone mass.

During the 20‐day PTH treatment, the osteoblastic microenvironment progressed through a differentiation cycle characterized by initial osteoblast proliferation, followed by osteoid accumulation and culminating in mineralization accompanied by a decline in osteoblast numbers. After 5 days of PTH treatment, the increase in osteoblast number was associated with an expanding calcein‐labeled area (Figure 2B–D), indicating progressive osteoblast differentiation. Correspondingly, osteoid and collagen secretion gradually increased beginning at 5 days posttreatment (Figure 2E), reflecting enhanced osteoblast activity and osteoid accumulation. After 10 days of treatment, osteoblast numbers peaked during the differentiation cycle (Figure 2C), and the distance between calcein and tetracycline fluorescence became apparent, suggesting an increased microscopic bone mineralization rate (Figure 2F). Von Kossa staining confirmed a significant increase in calcium deposition after 10 days of PTH treatment (Figure 2G). Although PTH induction for 15 days produces fewer osteoblasts than 10 days of induction, the cells became larger and more voluminous, indicating a stronger capacity for bone matrix secretion (Figure 2B,C). TRAP staining revealed osteoclast activation after 15 days of PTH treatment (Figure 2H). The mice treated with PTH for 20 days presented increased cortical bone mass (Ma.V↓; Figure 2I) and trabecular bone mass (BV/TV↑, Tb.Th↑; Figure 2J), as assessed by micro‐CT. Overall, on the basis of bone morphometric indicators, the PTH‐induced osteoblastic microenvironment can be divided into three phases (Figure 2K): the osteoid formation phase (0–10 days), the microscopic mineralization phase (10–20 days), and the CT‐visible mineralization phase (after 20 days).

To assess the bone colonization potential of cancer cells in different osteoblastic microenvironments, MDA231 RUNX2‐OE cells were inoculated intracardially into SCID mice that had received PTH for various durations. Mice treated with PTH for 10 days (PTH10) demonstrated multiorgan colonization in the bones, lungs, and lymph nodes, with a 75.0% (6/8) incidence of osteolytic lesions compared with 28.6% (2/7) in the control group (PTH0; Figure 2L–N). Correspondingly, tumor burden, the area of osteolytic lesions, and TRAP^+^ osteoclast surface were significantly greater in the PTH10 group than in the PTH0 group (Figure 2O,P). Given that osteoclasts were not activated in mice subjected to short‐term PTH treatment (less than 15 days; Figure 2B,H), the increase in osteolytic lesions in the PTH10 group of mice was attributed to tumor cell colonization in an unmineralized osteogenic microenvironment. The ability of MDA231 RUNX2‐OE cells to colonize the lung was weak in PTH0 mice (28.6%, 2/7) but increased to 87.5% (7/8) in the PTH10 group (Figure 2L; Figure S2A,B). Additionally, both subcutaneous and intrathoracic lymph node colonization were observed in PTH‐treated mice (Figure S2C–E). Although the lymph node colonization rates did not significantly differ from those in the PTH0 group (Figure S2E), the colonized lymph nodes exhibited noticeable swelling (Figure S2C). Lymph node colonization near the limbs resembled bone colonization on live imaging; however, clear distinctions were revealed upon dissection (Figure S2C,D). In summary, in combination with the bone morphometric phase, the osteoid formation phase, without further mineralization, contributes to multiorgan colonization by basal‐like MDA231 RUNX2‐OE cells.

### 2.3 Abnormally Activated Osteoblastic Microenvironments Reactivate Quiescent Basal‐Like Cancer Cells

**FIGURE 3:**
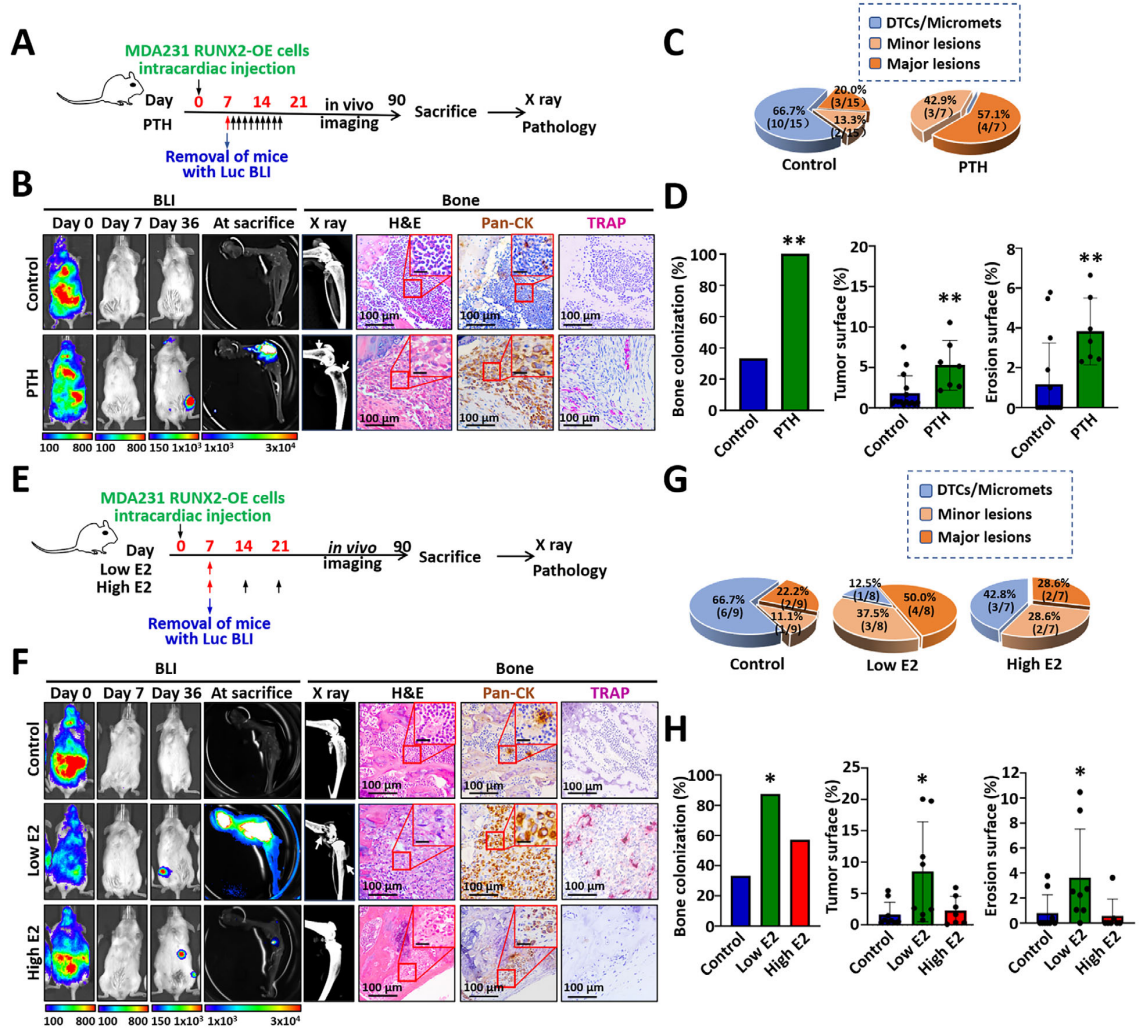
Abnormal activation of the osteogenic microenvironment reactivates quiescent basal‐like cancer cells. (A) Experimental schedule for PTH‐reactivated bone colonization. MDA231 RUNX2‐OE cells were injected into SCID mice via the left ventricle. The mice were then treated with 100 µg/kg PTH for 10 consecutive days. Mice inoculated with PBS served as controls. (B) Representative BLI, X‐ray, H&E staining, immunohistochemical staining for pan‐CK, and TRAP staining images demonstrating the progression of bone lesions and the activity of osteoclasts. (C) Pie charts displaying the incidence of DTCs/micrometastases and osteolytic lesions in mice treated with PTH and in control mice. (D) Bar charts showing the incidence of osteolytic bone colonization, tumor surface, and erosion surface in PTH‐treated mice and control mice. (E) Experimental schedule involving the injection of MDA231 RUNX2‐OE cells through the left ventricle, followed by treatment with either a single dose of low‐dose estradiol cypionate (E2, 0.3 mg/kg) or weekly high‐dose E2 (2 mg/kg) for 3 weeks via subcutaneous injection, starting 7 days post‐inoculation of cancer cells. Mice injected with an equal volume of the solvent corn oil served as controls. (F) Representative BLI, X‐ray, H&E staining, immunohistochemical staining for pan‐CK, and TRAP staining images illustrating the progression of bone lesions and the activity of osteoclasts. (G) Pie charts depicting the incidence of DTCs/micrometastases and osteolytic lesions in mice treated with various doses of E2. (H) Bar charts illustrating the incidence of osteolytic bone colonization, tumor surface, and erosion surface in mice subjected to low‐dose E2, high‐dose E2, and control conditions. The scale bars in the inset images indicate 20 µm. The data are presented as the means ± SDs. ^*^ *p* <0.05 and ^**^ *p* <0.01 compared with the control group, as determined by Fisher's exact probability method or Student's t test.

Considering the role of the unmineralized osteogenic microenvironment in promoting bone colonization, we investigated whether abnormal activation of the osteoblastic microenvironment could trigger reactivation of quiescent DTCs and micrometastases. We intracardially implanted MDA231 RUNX2‐OE cells into SCID mice. Early colonization was observed in approximately 23% of the mice that exhibited luciferase bioluminescence signals at 7 days post‐inoculation with tumor cells (Figure S3A,B). However, 90% of the mice that did not exhibit colonization at 7 days remained free of cancer cell colonization until 21 days post‐inoculation (Figure S3A,B). Therefore, in this study, mice exhibiting luciferase bioluminescence at 7 days post‐inoculation were removed from the study, while the remaining mice with quiescent micrometastases (Figure S3C) received daily doses of PTH or PBS (as controls) for 10 days to determine whether PTH could reactivate quiescent cancer cells (Figure 3A). MDA231 RUNX2‐OE cells remained quiescent in 66.7% (10/15) of the control mice, and these mice did not develop osteolytic lesions during the subsequent 3 months (Figure 3B–D). However, treatment with 10 doses of PTH, which induced the differentiation of an unmineralized osteogenic microenvironment, resulted in the development of osteolytic lesions in all the treated mice (100%, 7/7; Figure 3B–D). These findings suggest that an activated unmineralized osteogenic microenvironment can reactivate quiescent cancer cells and promote the formation of osteolytic lesions.

Estrogen plays a crucial role in regulating bone mass in adult women by promoting osteoblast differentiation and inhibiting osteoclast activity, and estrogen deficiency is a major cause of postmenopausal osteoporosis [21]. Given the link between estrogen fluctuations during perimenopause (ages 46 to 55) and an increased risk of bone metastasis (Figure S4A,B), our study investigated whether estrogen‐induced changes in the osteoblastic microenvironment reactivate quiescent, hormone‐independent MDA231 RUNX2‐OE cells and drive bone colonization and osteolytic lesions in mice. To simulate estrogen‐induced abnormal differentiation in the osteoblastic microenvironment, we administered a low dose of estradiol cypionate (E2), which resulted in an increase in the osteoblast number and collagen accumulation without altering mineralized bone mass, and a high dose of E2, which led to an increase in both osteoblast number and bone mass (Figure S4C–G). As shown in Figure 3E–H, low‐dose E2 reactivated quiescent MDA231 RUNX2‐OE cells and caused osteolytic lesions in 87.5% (7/8) of the mice, whereas high‐dose E2 did not significantly increase the incidence or size of the osteolytic lesions. These findings suggest that slight abnormalities in estrogen fluctuations during perimenopause may trigger a nonmineralized osteogenic microenvironment, leading to the reactivation of quiescent tumor cells and the promotion of osteolytic metastases. In contrast, a highly mineralized microenvironment associated with persistently elevated estrogen levels may hinder the survival and colonization of basal‐like cancer cells.

### 2.4 RUNX2 Promotes Bone Colonization by Luminal‐Like Breast Cancer Cells Within a Highly Mineralized Bone Microenvironment Supported by E2

**FIGURE 4:**
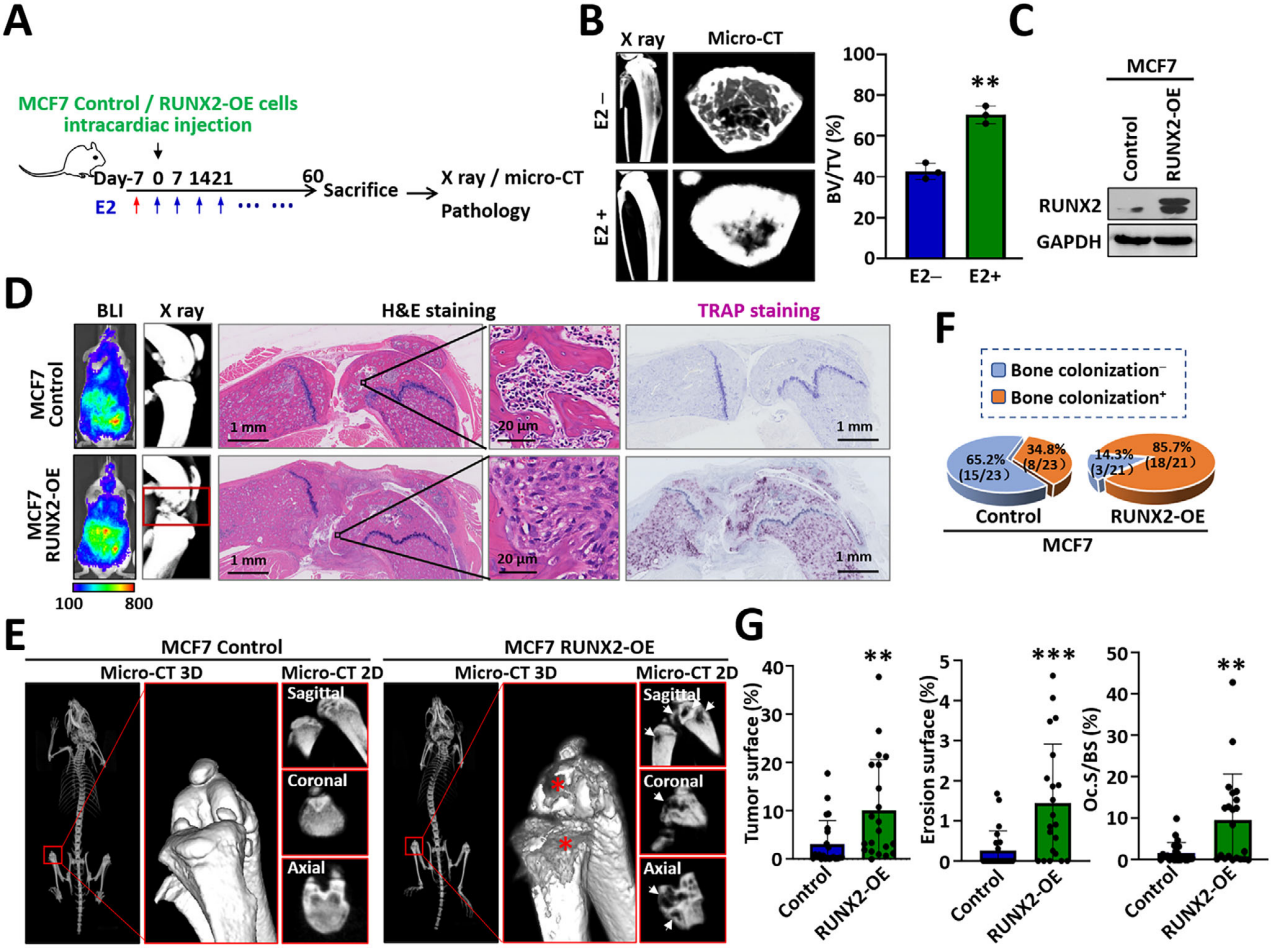
RUNX2 promotes the colonization of luminal‐like MCF7 cells within a highly mineralized osteogenic microenvironment induced by E2. (A) Experimental schedule for bone colonization of MCF7‐derived cells in SCID mice. MCF7 RUNX2‐OE cells and control cells were injected into the mice via the left ventricle. E2 (2 mg/kg) was administered via subcutaneous injection weekly to support MCF7 tumor growth, starting 1 week before tumor cell injection. (B) Representative micro‐CT and H&E staining images showing increased bone mass and a reduced marrow cavity in SCID mice administered E2. Bar graph illustrating the bone volume fraction (BV/TV) in mice treated with E2 compared with in control mice. (C) Western blot analysis of RUNX2 protein levels in MCF7 RUNX2‐OE cells and control cells. (D) Representative BLI images showing the systemic distribution of tumor cells following left ventricular inoculation on day 0. X‐ray and H&E staining images displaying bone lesions in mice after sacrifice. TRAP staining images indicating osteoclast activity. (E) Representative micro‐CT images displaying bone lesions in SCID mice on day 38. The osteolytic lesions are marked by asterisks and arrows. (F) Pie charts illustrating the incidence of osteolytic bone colonization in mice injected with MCF7 RUNX2‐OE cells compared with those in mice injected with control cells. (G) Bar charts showing the tumor surface, erosion surface, and TRAP^+^ surface normalized to the bone surface. The data are displayed as the means ± SDs. ^**^ *p* <0.01 and ^***^ *p* <0.001 compared to the control group, as determined by Student's t‐test.

MCF7‐derived cells, which are luminal‐like breast cancer cells, require estrogen support for tumor growth in mice (Figure 4A). Estrogen not only promoted the growth of MCF7 tumors but also activated osteoblasts and increased bone density. Micro‐CT and H&E staining revealed highly mineralized trabeculae and a reduced marrow cavity in E2‐treated mice (Figure 4B). In this highly mineralized bone microenvironment, 34.8% (8/23) of the control MCF7 cells with very low RUNX2 expression colonized the bones of the mice (Figure 4C–G). Conversely, osteolytic bone colonization of MCF7 RUNX2‐OE cells in 85.7% the mice (18/21), as detected by X‐ray and micro‐CT (Figure 4C–G). Owing to the high bone density caused by E2, the bone lesions formed by the MCF7 RUNX2‐OE cells detected on the X‐ray images, unlike the osteolytic lesions with a “moth‐eaten” appearance in homeostatic bone, exhibited growth through the bone cortex and outward (Figure 4D). However, micro‐CT clearly revealed osteolytic lesions within the bone (Figure 4E). Accordingly, the osteoclasts within the bone lesions were highly activated (Figure 4D,G). Overall, the increased incidence of bone colonization by MCF7 RUNX2‐OE cells highlights the strong collaboration between RUNX2‐OE luminal‐like cells and the highly mineralized active osteogenic microenvironment.

### 2.5 An Unmineralized Osteogenic Microenvironment Exacerbates Osteolytic Lesions Caused by Luminal‐Like Breast Cancer Cells

**FIGURE 5:**
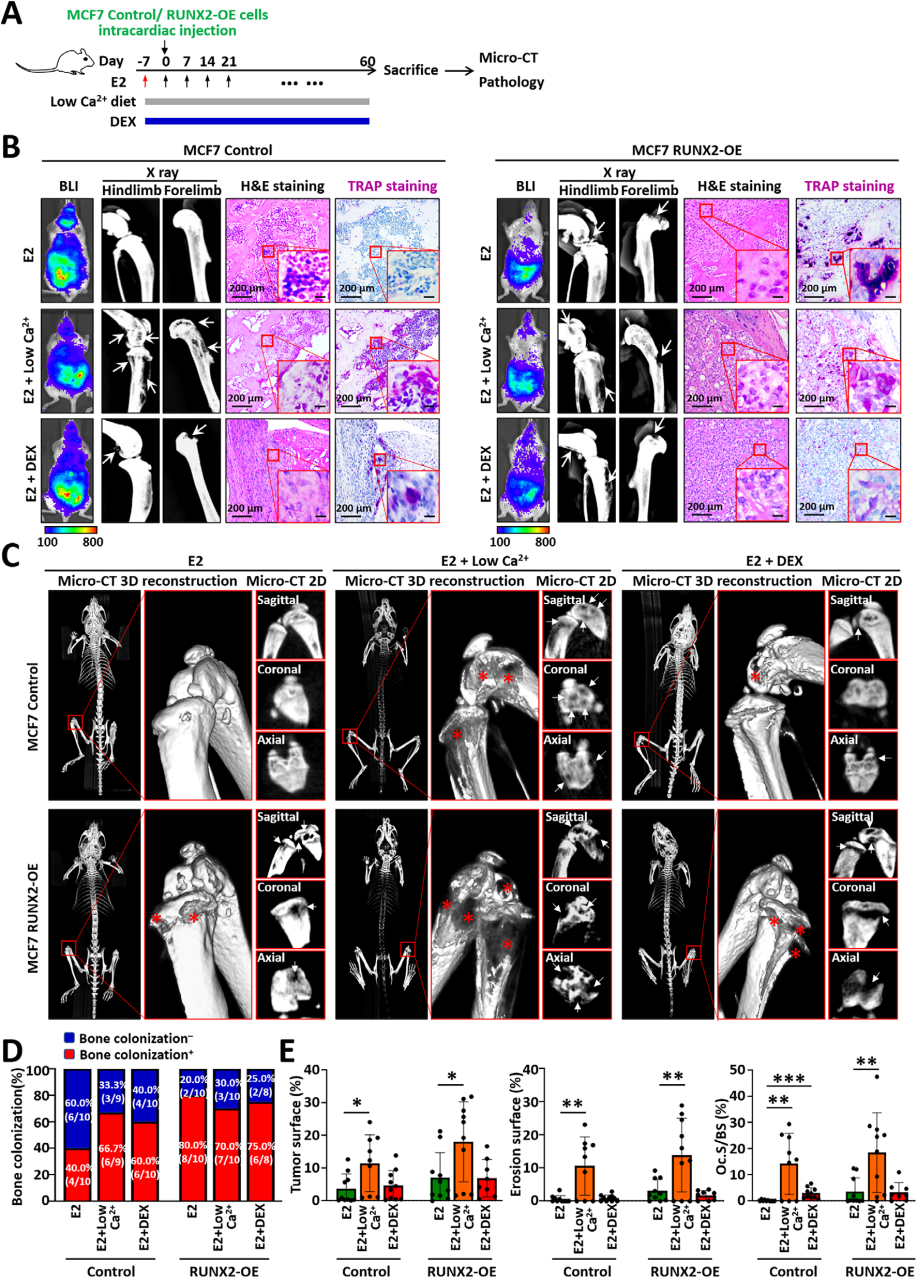
An unmineralized osteogenic microenvironment exacerbates osteolytic lesions caused by luminal‐like MCF7 cells. (A) Experimental diagram showing the bone colonization of MCF7‐derived cells within different osteogenic microenvironments established by the administration of E2. SCID mice were administered E2 (2 mg/kg) weekly, either alone or in combination with dexamethasone (DEX, 0.5 µg/mL in drinking water) or a low‐calcium diet. (B) Representative BLI images showing the systemic distribution of tumor cells following left ventricular inoculation on day 0. X‐ray and H&E staining images depict bone lesions in mice after sacrifice, whereas the TRAP staining images indicate osteoclast activity. (C) Representative micro‐CT images showing bone lesions in SCID mice on day 38. The osteolytic lesions are marked by asterisks and arrows. (D,E) Bar charts presenting the incidence of osteolytic bone colonization (D), tumor surface, erosion surface, and TRAP^+^ osteoclast surface (E). The scale bars in the inset images indicate 20 µm. The data are displayed as the means ± SDs. *p <0.05, ^**^ *p* <0.01 and ^***^ *p* <0.001 compared with the respective E2‐treated mice, as determined by Student's t test.

Given the inevitable activation of the osteoblastic microenvironment by E2, which is essential for the growth of luminal‐like cancer cells, we attempted to inhibit osteogenesis and mineralization by feeding a low‐calcium diet or administrating dexamethasone during E2 treatment (Figure 5A; Figure S5A). As shown in Figure S5B–E, the low‐calcium diet inhibited E2‐induced mineralization, resulting in a delay to an early mineralization stage and an overall decrease in bone mineral density, accompanied by increases in the osteoblast number and collagen accumulation, although localized increases in mineral density were observed. Moreover, dexamethasone effectively inhibited E2‐induced osteoblast differentiation and mineralization, leading to a modest increase in the amount of unmineralized collagen in E2‐treated mice without increasing the rate of trabecular bone mineralization, indicating the presence of an osteogenic microenvironment at the osteoid stage.

MCF7 RUNX2‐OE cells exhibited a high incidence of bone colonization in E2‐treated mice, whether the mice also received dexamethasone or were fed a low‐calcium diet, similar to the observations in mice treated with E2 alone (Figure 5B–E). However, control MCF7 cells exhibited a moderate increase in bone colonization only in mice fed a low‐calcium diet or in those treated with dexamethasone, compared with E2 treatment alone (Figure 5B–E). Notably, the accumulation of unmineralized osteoids induced by the low‐calcium diet dramatically exacerbated bone lesions originating from both MCF7 control and RUNX2‐OE cells and was accompanied by robust osteoclast activation, whereas dexamethasone treatment resulted in minor lesions (Figure 5B–E). Together, these findings indicate that an unmineralized osteogenic microenvironment, characterized by osteoid accumulation, promotes the expansion of osteoclastic lesions driven by luminal‐like cancer cells.

### 2.6 Osteoclasts Can be Activated By Tumor Cells and Well‐Differentiated Osteoblasts

Since breast cancer patients frequently experience osteolytic bone metastases, we investigated the interactions between osteoclasts and tumor cells, and their relationship with the osteogenic microenvironment, via an in vitro coculture system (Figure S6A). Our results revealed that mOC progenitors were activated by both MDA231 and MCF7 breast cancer cells (Figure S6B,C). Additionally, while preosteoblast MC3T3‐E1 cells did not trigger osteoclast activation, well‐differentiated MC3T3‐E1 cells induced by osteogenic media or PTH significantly activated osteoclasts (Figure S6B,C). Overall, these results provide insight into how breast cancer cells colonizing in an osteogenic microenvironment activate osteoclasts, thereby contributing to the formation of osteolytic lesions.

### 2.7 Osteoblasts and the Bone Matrix Within Osteogenic Microenvironment at Various Differentiation Stages Have Distinct Effects on the Chemotaxis and Proliferation of Cancer Cells

**FIGURE 6:**
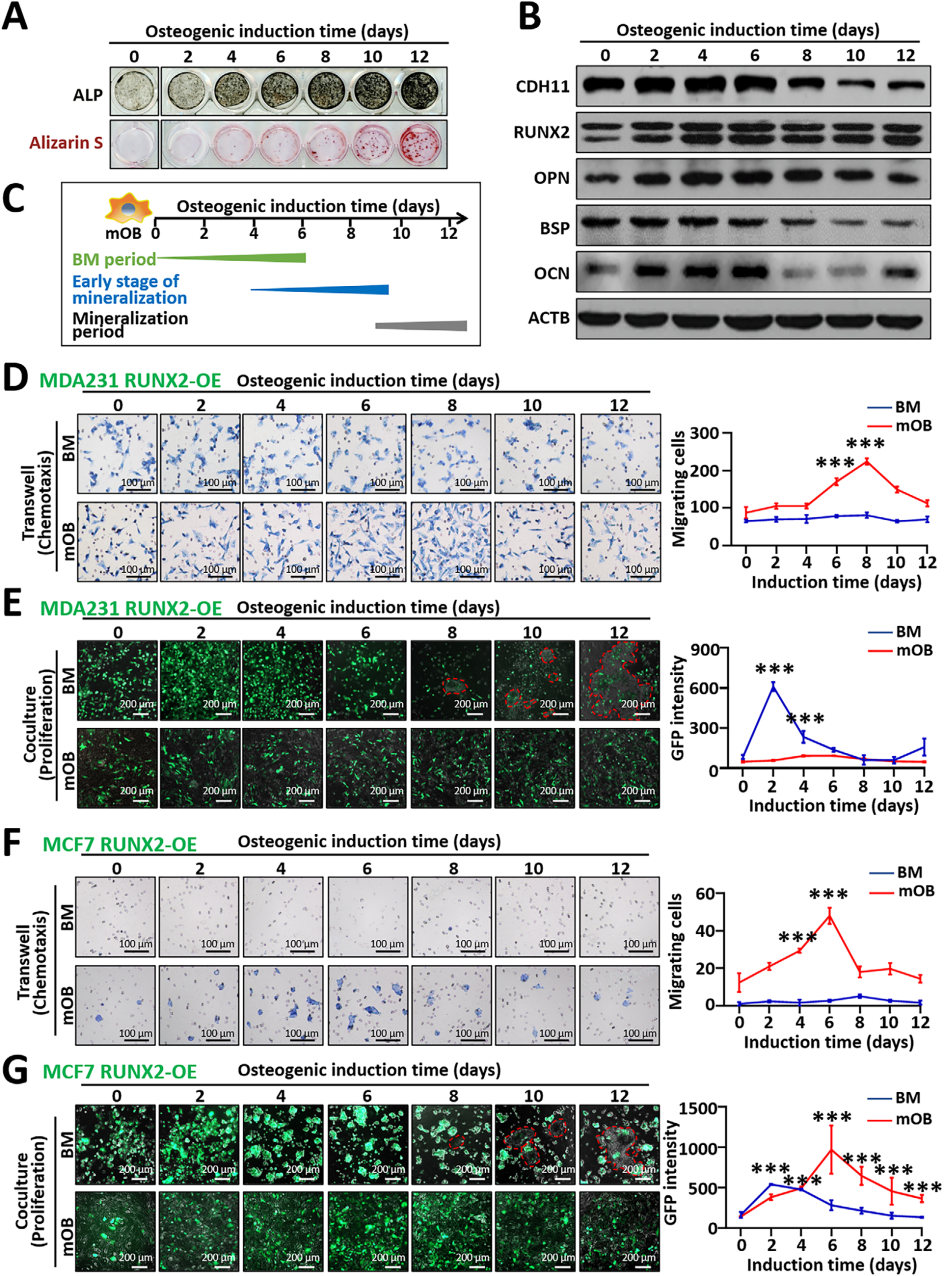
Osteoblasts and the bone matrix exhibit distinct effects on RUNX2‐overexpressing breast cancer cells in different osteoblastic microenvironments in vitro. Mouse primary osteoblasts (mOBs) were induced in osteogenic media supplemented with 50 µg/mL L‐ascorbic acid and 10 mm β‐glycerophosphate disodium for 0, 2, 4, 6, 8, 10, or 12 days. mOBs were isolated by digesting the cells with 0.25% trypsin‐EDTA, while the bone matrix (BM) was obtained by removing the cell components by treatment with 20 mm NH_4_OH and 0.5% Triton X‐100 for 5 min. (A) Representative ALP staining and alizarin S staining images after different durations of osteogenic induction. (B) Western blot analysis of osteogenesis‐related protein levels. (C) Staging of the osteoblastic microenvironment in vitro on the basis of various induction durations. (D,E) Effects of mOBs and BM at different stages of differentiation on the chemotactic (D) and proliferative (E) capacities of GFP‐labeled MDA231 RUNX2‐OE cells. Calcium nodules are circled with red dashed lines. (F,G) Effects of mOBs and BM at different stages of differentiation on the chemotactic (F) and proliferative (G) capacities of GFP‐labeled MCF7 RUNX2‐OE cells. Calcium nodules are circled with red dashed lines. The data are presented as the means ± SDs. ^***^ *p* <0.001 compared with the respective control group (0 days), as determined by Student's t‐test.

To investigate the specific effects of osteoblasts and the bone matrix on cancer cells, we induced primary mouse osteoblasts (mOBs) into various differentiation states by varying the duration of osteogenic induction in vitro. As shown in Figure 6A,B, ALP activity increased with increasing duration of osteogenic induction, accompanied by elevated levels of osteogenesis‐related proteins from days 2 to 6. Calcium nodules appeared by day 4, with more prominent calcified nodules observable by day 10. Consequently, the in vitro osteoblastic microenvironment was classified into three stages (Figure 6C): the bone matrix (BM) period (0–6 days), the early mineralization stage (4–10 days), and the mineralization period (after 10 days).

We then separated osteoblasts and the bone matrix from the osteogenic cultures and evaluated the migration of cancer cells toward osteoblasts or the bone matrix, as well as their proliferation when they were cocultured with either osteoblasts or the bone matrix. Osteoblasts after 6 to 8 days of osteogenic induction (mOB6 and mOB8) were significantly more attracted to basal‐like MDA231 RUNX2‐OE cells than were control osteoblasts (mOB0). However, this chemotactic response diminished with increasing induction duration (Figure 6D). Bone matrix acquired after osteogenic induction for different durations did not significantly affect cancer cell migration but had a considerable effect on the proliferation of MDA231 RUNX2‐OE cells. Cell proliferation was notably greater in the unmineralized bone matrix after 2 to 4 days of induction, whereas the mineralized bone matrix inhibited proliferation (Figure 6E). Control MDA231 cells exhibited a trend similar to that of the RUNX2‐OE cells; however, the effects of changes in the osteoblastic microenvironment on control cells were significantly weaker (Figure S7A,B). Therefore, during the early stage of mineralization, osteoblasts attract basal‐like cancer cells, while the mineralized matrix hinders their proliferation, potentially resulting in quiescent micrometastases in the bone marrow.

We further investigated the specific effects of osteoblasts and the bone matrix at different differentiation states on luminal‐like MCF7 cancer cells in vitro (Figure 6F,G; Figure S7C,D). Well‐differentiated osteoblasts induced for 4 to 8 days demonstrated robust recruitment of both MCF7 RUNX2‐OE cells and control cells (Figure 6F; Figure S7C). Unmineralized fresh bone matrix from days 2 to 4 of differentiation increased the proliferation of the MCF7 RUNX2‐OE cells (Figure 6G), similar to the response observed in basal‐like MDA231 cells. Unlike MDA231 RUNX2‐OE cells, however, MCF7 RUNX2‐OE cells retained a proliferative advantage with support from osteoblasts throughout the mineralization process (Figure 6G). The effects of the bone matrix and osteoblasts on the proliferation of control MCF7 cells were qualitatively similar yet attenuated compared with their effects on the proliferation of MCF7 RUNX2‐OE cells (Figure S7D). Together, these findings suggest that during early mineralization, mature osteoblasts attract luminal‐like cancer cells, providing them with a survival advantage in bone, even when the mineralized matrix is less conducive for colonization. This interpretation is consistent with clinical [22] and experimental [23, 24] evidence justifying the increased risk of bone metastasis associated with luminal‐like cancer cells.

### 2.8 Cell–Cell Junctions and Cell–Matrix Adhesion Underlie the Differential Interactions of Luminal and Basal‐Like Cancer Cells With the Osteoblastic Microenvironment

The transcriptional profiles of RUNX2‐overexpressing MDA231 and MCF7 cells, as well as their respective control cells, were investigated using RNA sequencing. The transcriptional profiles of the MDA231‐derived cells differed from those of the MCF7‐derived cells (Figure S8A; Tables S1 and S2), and involved metastasis‐related processes, including the estrogen response, tumor necrosis factor α (TNF‐α) signaling, epithelial–mesenchymal transition (EMT), hypoxia, the interferon (IFN) response, apical junctions, and the WNT signaling pathway (Figure S8B). Given the distinct effects of osteoblasts on the proliferation of MCF7‐derived and MDA231‐derived cells, we further performed a detailed analysis of genes related to cell–cell junctions and cell–matrix adhesion. As shown in Figure S8C,D, genes related to cell–matrix adhesion were enriched in basal‐like MDA231‐derived cancer cells, whereas luminal‐like MCF7‐derived cancer cells exhibited greater enrichment of molecules related to cell–cell junctions. These results indicate a close interaction between luminal‐like cancer cells and osteoblasts, highlighting the supportive mechanism through which active osteoblasts drive luminal‐like cancer cell proliferation.

We also identified genes regulated by RUNX2 in MDA231 and MCF7 cells and validated the key genes regulated by RUNX2 using RT‐qPCR (Tables S3–S5; Figure S8E–H). RUNX2 overexpression in these cancer cells altered the expression of genes involved in the cell cycle, adhesion, migration, apoptosis, and other metastasis‐related biological processes (Figure S8E–H). Notably, RUNX2 overexpression in MDA231 cells led to upregulation of genes associated with osteoblast differentiation and ossification (Figure S8G,H), indicating an enhanced “osteomimetic” phenotype that facilitates cancer cell survival in the osteoblastic microenvironment. Specifically, RUNX2 also increased the expression of hypoxia‐related genes (Figure S8G,H), suggesting greater responsiveness to marrow hypoxia, therefore promoting cancer cell survival and colonization in bone. Furthermore, RUNX2 activated the canonical NF‐κB signaling pathway (Figure S8G) and suppressed the interferon response (Figure S8H), which resulted in osteoclast activation and the formation of osteolytic lesions. Together, the RUNX2‐driven changes in gene expression collectively promote breast cancer cell recruitment, adhesion, and proliferation within the osteogenic microenvironment, as well as osteoclast activation and osteolysis.

### 2.9 Blocking the Interaction Between Breast Cancer Cells and the Bone Matrix Reduces Bone Colonization

**FIGURE 7:**
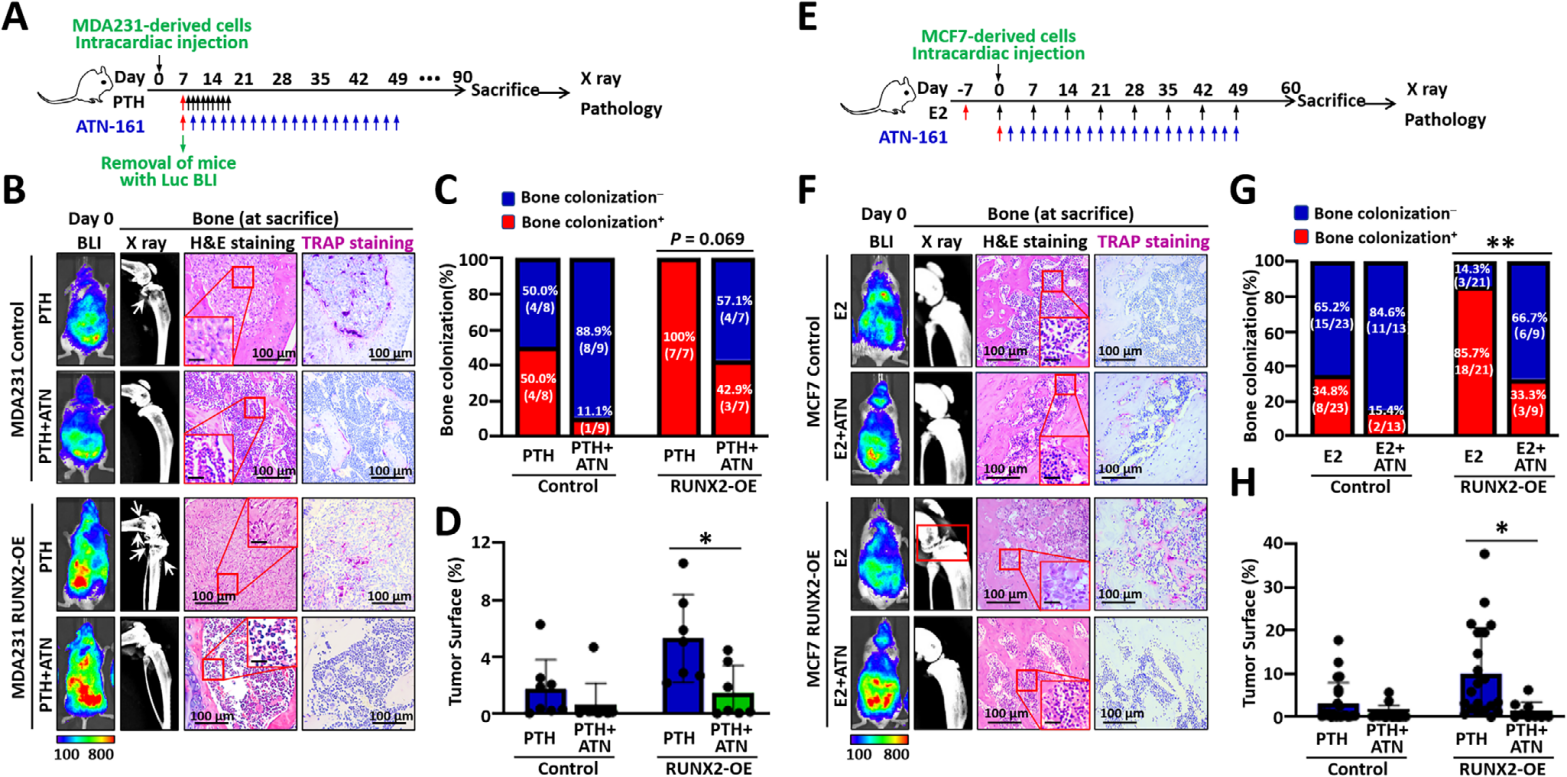
The ITGA5 antagonist ATN‐161 reduces the incidence of bone colonization. (A–D) Experimental diagram (A), representative X‐ray, H&E staining, TRAP staining, and KI67 and pan‐CK immunofluorescence staining images (B), and bar charts demonstrating the inhibition of PTH‐reactivated bone colonization (C) and tumor surface normalized to the bone surface (D), in MDA231‐derived cells following ATN‐161 administration. (E–H) Experimental diagram (E), representative X‐ray, H&E staining, TRAP staining, and KI67 and pan‐CK immunofluorescence staining images (F), and bar charts displaying the blockade of E2‐supported bone colonization (G) and tumor surface normalized to the bone surface (H), in MCF7‐derived cells after ATN‐161 administration. The scale bars in the inset images indicate 20 µm. The data are presented as the means ± SDs. *p <0.05, ^**^ *p* <0.01 compared with the respective control mice, as determined by Fisher's exact probability test or Student's t test.

Considering the evidence that an unmineralized bone matrix supports the proliferation of both basal‐like and luminal‐like cancer cells, along with our previous finding that ITGA5 mediates the interaction between tumor cells and the bone matrix [10], we employed ATN‐161, an antagonist of ITGA5, to investigate whether disrupting this interaction could further reduce bone colonization. We found that ATN‐161 decreased the incidence of bone colonization by both basal‐like MDA231 RUNX2‐OE cancer cells activated by PTH treatment (Figure 7A–D) and luminal‐like MCF7 RUNX2‐OE cancer cells in the highly mineralized osteoblastic microenvironment induced by E2 (Figure 7E–H). Thus, targeting the interaction between tumor cells and the bone matrix represents a promising strategy to prevent the reactivation of quiescent cancer cells and subsequent bone colonization.

## 3 Discussion

The effect of the osteoblastic microenvironment on bone colonization by breast cancer cells remains debated. Two studies conducted by Wang H and colleagues suggested that the osteogenic microenvironment provides a “supportive” niche for breast cancer bone colonization and metastasis [5, 23]. On the one hand, the activation of the mTOR signaling pathway via heterotypic adherens junctions involving cancer‐derived E‐cadherin and osteogenic N‐cadherin drives the progression from single cells to micrometastases in the bone [5]. On the other hand, the osteogenic niche promotes cancer cell proliferation and the progression of bone metastasis through the delivery of calcium ions to tumor cells via gap junctions [23]. However, Pradhan L and colleagues [25] reported that osteoblasts inhibit bone colonization by ER^+^ breast cancer cells by inducing cancer cell dormancy and autophagy. Capulli M. et al. [26] identified a specific N‐cadherin^+^/CD45^−^ osteoblast subpopulation lining the endosteal surface of the trabecular bone that induces breast cancer cell quiescence through the Notch/Jagged pathway. Therefore, a comprehensive explanation for the divergent outcomes of cancer cells, specifically whether they settle as DTCs/micrometastases or develop into bone metastases, is lacking. In this study, we induced the osteoblastic microenvironment to differentiate into various states in vitro and in vivo and investigated how the differentiation status of the osteoblastic microenvironment affects the fate of cancer cells, focusing on whether these cells colonize the bone, enter a quiescent state as DTCs or micrometastases, or reactivate from dormancy.

**FIGURE 8:**
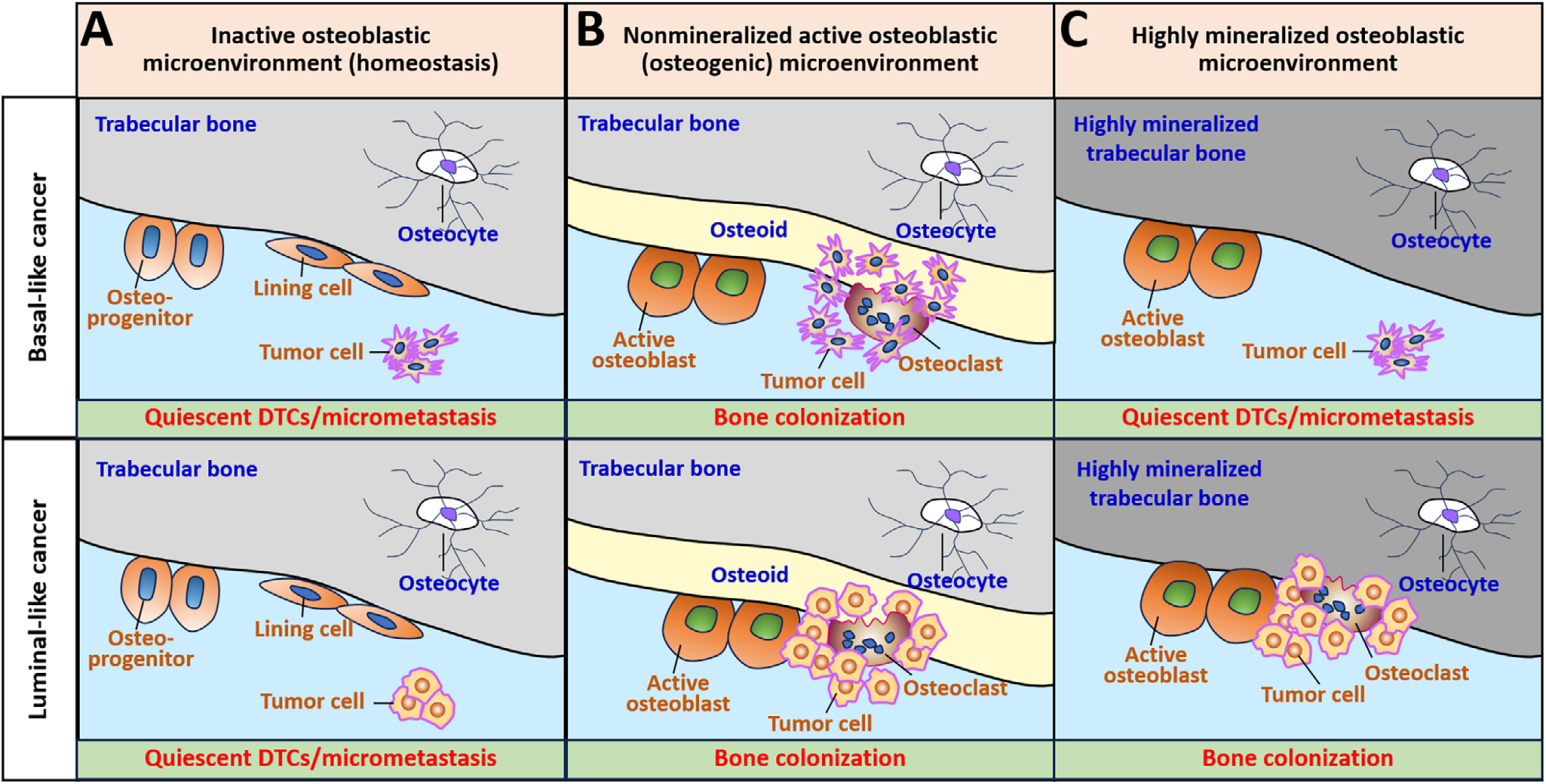
Schematic illustration of how the osteoblastic microenvironment determines the fate of breast cancer cells disseminated in the bone marrow.

Under normal physiological conditions in adults, bone homeostasis reflects an inactive osteoblastic microenvironment maintained by a delicate balance between osteogenesis and osteolysis. In this context, the osteoblastic microenvironment consists of mineralized bone matrix and inactive osteoblasts, which primarily exist as inactive lining cells and progenitors, along with terminally differentiated osteocytes embedded in the bone matrix [27]. In this study, basal‐like cancer cells were observed to remain quiescent as DTCs or micrometastases in homeostatic bone in mice, and in vitro experiments confirmed their slow proliferation in an inactive osteoblastic microenvironment. While we cannot replicate the state of luminal‐like cancer cells in murine homeostatic bone because of the inevitable activation of the osteoblastic microenvironment by E2, which is essential for cancer cell growth, luminal‐like cells similarly exhibit slow proliferation under inactive osteoblastic conditions in vitro. Collectively, these in vivo and in vitro findings suggest that tumor cells that disseminate into an inactive osteoblastic microenvironment during homeostasis often enter quiescence as DTCs or micrometastases (Figure 8A).

An active, nonmineralized, well‐differentiated osteoblastic (osteogenic) microenvironment, characterized by the accumulation of unmineralized osteoids, has been shown to support the survival and proliferation of both metastatic basal‐like and luminal‐like cancer cells (Figure 8B). This nonmineralized osteogenic microenvironment may arise from cytokines or extracellular vesicles released by tumor cells (premetastatic niche) [13], abnormal osteoblast metabolism, or fluctuating estrogen levels during perimenopause. Furthermore, abnormally activated nonmineralized osteogenic microenvironments reactivate quiescent DTCs and micrometastases, ultimately leading to osteolytic bone metastasis.

An active, highly mineralized osteogenic microenvironment affects basal‐like and luminal‐like breast cancer cells differently. When faced with challenges in highly mineralized bone, basal‐like cancer cells may remain quiescent as DTCs or micrometastases (Figure 8C). In contrast, luminal‐like cancer cells tend to survive with the support of active osteoblasts and are capable of colonizing bone (Figure 8C). The survival advantage of luminal‐like cancer cells in a highly mineralized osteogenic microenvironment independent of osteoids, offers an explanation for the clinical observation that luminal‐like cancers have a higher risk of bone metastasis, despite the similar prevalence of bone marrow DTCs in both cancer subtypes [28, 29].

While the colonization of cancer cells in bone depends on the differentiation status of the osteoblastic microenvironment, established tumor colonies can subsequently activate osteoclasts, resulting in the formation of osteolytic lesions. During the process of bone colonization, tumor cells secrete cytokines such as parathyroid hormone‐related protein (PTHrP), receptor activator of NFκB ligand (RANKL), and interleukins (ILs), which stimulate osteoclastogenesis and lead to localized bone resorption. Increased osteoclast activity results in the release of growth factors, including transforming growth factors (TGFs), insulin‐like growth factors (IGFs), platelet‐derived growth factor (PDGF), and bone morphogenetic proteins (BMPs), thereby promoting tumor growth and further osteolytic factor secretion. The reciprocal interaction between tumor cells and osteoclasts has been extensively characterized as a “vicious cycle” [30, 31]. Furthermore, breast cancer cells with high bone metastatic potential can induce osteogenic differentiation in the early stage of bone colonization [13, 24, 32]. Well‐differentiated osteoblasts, which produce macrophage colony‐stimulating factor (M‐CSF) and RANKL [33], contribute to bone resorption when tumor cells disrupt bone homeostasis.

While PTH(1‐34) has been reported to stimulate both bone formation and resorption, thereby increasing bone turnover [18], our study revealed that low concentrations of PTH(1‐34) applied for a short duration do not directly activate osteoclasts. Instead, PTH(1‐34) appears to stimulate osteoclast activation indirectly through activated osteoblasts. Conflicting results have been reported regarding the effect of PTH(1‐34) on breast cancer cell bone colonization. Several studies have demonstrated that 4 weeks of treatment with PTH(1‐34) increases bone volume and reduces breast cancer growth within the bone [34, 35], whereas another study indicated that a short‐term (5 days) pretreatment with PTH(1‐34) leads to an increase in tumor colonies throughout skeletal sites [36]. Our study revealed that the impact of PTH(1‐34) on breast cancer cell bone colonization varies depending on the osteoblastic microenvironment. Specifically, a highly mineralized microenvironment resulting from long‐term PTH(1‐34) treatment hinders basal‐like breast cancer colonization, whereas unmineralized osteoids resulting from short‐term treatment increase bone colonization. These findings highlight the necessity for regular long‐term medication administration and monitoring of bone metastasis‐related markers in breast cancer patients receiving bone‐modifying agents.

Estrogen is a crucial endocrine factor that regulates the differentiation status and activity of osteoblasts [37]. Estrogen receptor (ER) alpha in osteoblastic lineage cells mediates the protective effect of estrogen against bone loss [38, 39]. In perimenopausal breast cancer patients with fluctuating estradiol levels [40], the osteoblastic microenvironment may be temporarily activated but not sufficiently activated to support mineralization, potentially explaining the increased incidence of bone metastases in these patients. In patients with luminal‐like breast cancer, estrogen not only promotes the growth of cancer cells but also induces differentiation of the osteoblastic microenvironment, creating a favorable “soil” for bone metastasis.

This study clarified the distinct roles of osteoblasts and the bone matrix in the bone colonization of breast cancer cells. In general, well‐differentiated osteoblasts attract tumor cells, whereas the unmineralized bone matrix is primarily responsible for providing a supportive niche for cancer cell settlement and growth. Well‐differentiated osteoblasts secrete numerous cytokines [41, 42], potentially contributing to the significant increase in tumor cell recruitment. Once they reach the bone, cancer cells can either colonize or enter a quiescent state, which is determined by the level of mineralization in the bone matrix. An abundant unmineralized bone matrix facilitates cancer cell survival through its interaction with ITGA5 on the surface of tumor cells, as demonstrated in our previous study [10]. Furthermore, a newly formed poorly mineralized bone matrix with few collagen cross‐links has been observed in bone metastasis biopsies from breast cancer patients, supporting the role of a nonmineralized bone matrix in bone colonization [32]. Conversely, a highly mineralized bone matrix hinders cancer cell proliferation while allowing long‐term latency. Additionally, experimental evidence from Choi S et al. [43] suggests that the mineralization of the bone matrix dampens integrin‐mediated mechanosignaling, resulting in a less proliferative stem‐cell‐like phenotype associated with dormant DTCs. Among the integrin family members, ITGA5 is most closely associated with bone metastasis in the context of breast cancer [10, 44, 45]. Thus, we propose that the absence of ITGA5‐mediated growth signaling in a highly mineralized microenvironment triggers cancer cell dormancy.

ATN‐161 is a five‐amino acid peptide derived from the synergy region of fibronectin and functions as an antagonist of integrin α5β1 [46, 47]. ATN‐161 has been investigated as an anti‐angiogenic and anti‐tumor agent, which inhibits tumor cell invasion, proliferation, and angiogenesis. In the bone microenvironment, ATN‐161 disrupts the interaction between integrins and bone matrix proteins by competitively binding to the ITGA5 subunit, thereby preventing the reactivation of quiescent cancer cells and subsequent bone colonization.

RUNX2 plays a dual role in both breast cancer cells and osteoblasts during the process of bone metastasis. As an essential transcription factor for bone development and osteoblast maturation, RUNX2 is often overexpressed in breast cancer cells with high bone metastatic potential [48]. In breast cancer cells, RUNX2 facilitates bone metastasis by driving the expression of osteogenic‐related genes, resulting in an “osteomimetic” phenotype [49]. Our previous study revealed that ITGA5 is a transcriptional target of RUNX2 and a key mediator of RUNX2‐driven bone metastasis [10]. The ITGA5‐mediated interactions between cancer cells and the bone matrix, along with osteomimetic features, promote cancer cell migration toward bone, increase adhesion to the bone matrix, and provide a survival advantage within the osteoblastic microenvironment [10]. Additionally, RUNX2 derived from tumor cells increases RANKL expression [50] and activates the NF‐κB signaling pathway, leading to osteoclast activation and subsequent osteolytic lesions. On the other hand, the increase in RUNX2 expression in osteoblasts suggests an active osteoblastic microenvironment, creating a favorable metastatic niche for breast cancer cells by stimulating the production of osteoids. Furthermore, our earlier work [13] demonstrated that tumor‐derived RUNX2 increases the production of CDH11^high^/ITGA5^high^ EVs, contributing to the formation of an osteogenic premetastatic niche and facilitating bone colonization. Therefore, both tumor cell‐derived and osteoblast‐derived RUNX2 establish the “seed” and “soil” context essential for breast cancer bone metastasis.

Serum bone resorption markers have been used as diagnostic indicators for osteolytic metastasis in patients with breast cancer [51], whereas serum bone formation markers, such as ALP [52, 53] and the N‐terminal propeptide of type‐1 collagen (P1NP) [54, 55], have been shown to predict the development of bone metastasis in the short‐term. Notably, elevated serum P1NP levels specifically indicate an increased risk of short‐term bone metastasis in patients with stage I‐III breast cancer [54, 55], highlighting the importance of monitoring bone turnover markers during long‐term follow‐up, particularly in perimenopausal patients. In response to the osteogenic microenvironment, targeting the integrin signaling pathway may offer promising strategies for preventing the reactivation of quiescent cancer cells and bone colonization.

## 4 Experimental Section

### 4.1 Cells

The human breast cancer cell lines MDA‐MB‐231 (MDA231; RRID: CVCL_0062) and MCF7 (RRID: CVCL_0031), the mouse breast cancer cell line 4T1, and the mouse osteoblast progenitor cell line MC3T3‐E1 (RRID: CVCL_0409) were obtained from the American Type Culture Collection (ATCC). MDA231 and 4T1 cells (RRID: CVCL_0125) were maintained in RPMI 1640 medium (Thermo Fisher, 11875093) supplemented with 10% fetal bovine serum (FBS; Thermo Fisher, 10270106); MCF7 cells were cultured in DMEM (Thermo Fisher, 11995065) supplemented with 10% FBS; and MC3T3‐E1 cells were cultured in α‐MEM (Thermo Fisher, 12571063) supplemented with 10% FBS. All the cell lines were authenticated by short tandem repeat profiling and tested for mycoplasma contamination.

Primary mOBs were isolated from the cranial bones of newborn BALB/c mice (GemPharmatech, RRID:IMSR_GPT: N000020) as described previously [13] and cultured in α‐MEM supplemented with 10% FBS. Primary mOBs were identified by alkaline phosphatase (ALP) staining (Solarbio, G1481) and alizarin S staining (Solarbio, G1450) after being cultured in osteogenic media, which consisted of α‐MEM with 10% FBS, 50 µg/mL L‐ascorbic acid (Sigma–Aldrich, A92902), and 10 mM β‐glycerophosphate disodium (Sigma–Aldrich, A4544), for 12 days.

### 4.2 Lentiviral Construction and Infection

Lentiviruses containing fusions of a 3×Flag tag with either full‐length human *RUNX2* or mouse *Runx2*, both of which have a green fluorescent protein (GFP) reporter driven by a separate cytomegalovirus (CMV) promoter, were constructed. Breast cancer cells were infected with these lentiviruses and the corresponding control lentiviruses as previously described [13].

### 4.3 In Vitro Osteogenic Differentiation and Isolation of Osteoblasts and the Bone Matrix

To create various osteoblastic microenvironments, mOBs were cultured in osteogenic media for different durations (0, 2, 4, 6, 8, 10, and 12 days). Osteoblasts were then isolated by digesting the cells with 0.25% trypsin‐EDTA (Thermo Fisher, 25200072), or the bone matrix was obtained by incubating the cultures in 20 mm NH_4_OH and 0.5% Triton X‐100 (Sigma–Aldrich, 93443) for 5 min to remove cell components.

### 4.4 Chemotactic Migration Assay

The chemotactic migration of breast cancer cells was evaluated using 8‐µm pore Transwell inserts (BD Biosciences, 303597). Osteoblasts or bone matrix in various differentiation stages were placed in the lower chambers to attract cancer cells (2.5 × 10^4^) from the upper chambers for 13 h (MDA231 cells) or 18 h (MCF7 cells). The migrated cells were stained with crystal violet (Beyotime, C0121) and counted under a microscope. MDA231‐derived cells were counted in 6 random fields across 3 independent experiments (cells/field), while the total number of MCF7‐derived cells migrating through the membrane was counted in 3 independent experiments (cells/well).

### 4.5 Proliferation Assay

GFP‐labeled cancer cells were seeded in 24‐well plates (2.5 × 10^4^ cells/well) precoated with osteoblasts or bone matrix at various differentiation stages. After 72 h, the number and fluorescence intensity of GFP^+^ cells in 3 random microscope fields were determined to assess the proliferation of cancer cells in various osteoblastic microenvironments.

### 4.6 In Vitro Osteoclast Differentiation Assay

Mouse primary osteoclasts (mOCs) were differentiated from bone marrow‐derived monocytes isolated from 6‐week‐old C57BL/6 mice (GemPharmatech, RRID:IMSR_GPT: N000013). Initially, monocytes were seeded in a 24‐well plate (5 × 10^5^ cells/well) and induced to differentiate into mOC progenitors by treatment with M‐CSF (50 ng/mL; PeproTech, 315‐02) for 2 days. mOC progenitors were subsequently cultured in media supplemented with RANKL (50 ng/mL; PeproTech, 315‐11) for an additional 5 days. Alternatively, mOC progenitors were cocultured with cancer cells (MDA231 or MCF7) and preosteoblast cells (MC3T3‐E1) using Transwell coculture inserts (BD Biosciences). In the coculture system, mOC progenitors were cultured in the lower chamber in the presence of 50 ng/mL RANKL, while 1 × 10^4^ cancer cells or preosteoblast MC3T3‐E1 cells were seeded in the upper chamber. To evaluate the effect of osteoblast differentiation status on osteoclast activity, MC3T3‐E1 cells in the upper chamber were induced with osteogenic media or 0.5 ng/mL PTH(1‐34) (MCE, HY‐P0059). Moreover, mOCs treated with osteogenic media or PTH(1‐34) alone were used to evaluate any potential effects on osteoclast activity caused directly by the reagents. Tartrate‐resistant acid phosphatase (TRAP) staining (Solarbio, G1492) was used to detect mature osteoclasts.

### 4.7 Western Blotting

Western blotting was performed using the following primary antibodies: mouse anti‐RUNX2 (RRID: AB_2892645), rabbit anti‐bone sialoprotein (BSP; RRID: AB_725746), rabbit anti‐cadherin 11 (CDH11; RRID: AB_10547881), rabbit anti‐osteocalcin (OCN; RRID: AB_10675660), rabbit anti‐osteopontin (OPN; RRID: AB_3677274), mouse anti‐glyceraldehyde‐3‐phosphate dehydrogenase (GAPDH; RRID: AB_561053), and mouse anti‐actin beta (ACTB; RRID: AB_2242334). The bands were visualized with an enhanced chemiluminescence detection system (Promega, Madison, WI, USA).

### 4.8 Principles for Animal Experiments

The incidence of bone colonization by breast cancer cells and the presence of DTCs/micrometastases in bone marrow were investigated in mice with various osteoblastic microenvironments. The experimental endpoints were when the tumor reached 20 mm in diameter for a single tumor or 15 mm for multiple tumors, when there was 20% weight loss, signs of significant stress, more than 20% necrosis or ulceration, or experimental termination occurred. Bone colonization was identified via X‐ray imaging, micro‐CT imaging, and H&E staining, whereas DTCs/micrometastases were detected via immunohistochemical staining for pan cytokeratin (pan‐CK). The tumor burden was quantified as the percentage of tumor surface normalized to the total bone surface, as determined by pan‐CK immunohistochemical staining. TRAP staining was used to identify mature osteoclasts in metastatic bone lesions, and activity was quantified by the number of TRAP^+^ osteoclasts normalized to the bone surface (N.Oc/BS) or the TRAP^+^ osteoclast surface normalized to the bone surface (Oc.S/BS). All experiments were conducted in accordance with animal ethics guidelines and were approved by the Animal Ethics Committee of Tianjin Medical University Cancer Institute and Hospital (Ethical approval No. AE‐2022046).

### 4.9 Induction of an Osteogenic Premetastatic Niche In Vivo

A PMN was initiated in 4‐week‐old female BALB/c mice by injecting 4T1‐derived CDH11^high^/ITGA5^high^ extracellular vesicles into via the tail vein over 2 weeks or in 4‐week‐old NOD‐SCID mice (GemPharmatech, RRID:IMSR_GPT: T001492) by injection of MDA231‐derived CDH11^high^/ITGA5^high^ extracellular vesicles via the tail vein over 3 weeks, as previously described [13].

### 4.10 Induction of Various Osteoblastic Microenvironments In Vivo

Various osteoblastic microenvironments were induced either by varying the duration of PTH treatment or by administering E2 in combination with dexamethasone administration or by feeding a low‐calcium diet to investigate basal‐like cells and luminal‐like cells, respectively. To establish various PTH(1‐34)‐induced osteoblastic microenvironments, 6‐week‐old female NOD‐SCID mice received daily intraperitoneal injections of 100 µg/kg PTH for 0, 5, 10, 15, or 20 days. The mice in the 20‐day group were treated with PTH(1‐34) first. Five days later, injection began in the 15‐day group, followed by the 10‐day group after an additional five days, and the 5‐day group after an additional five days. The PTH injections in each group were completed on the same day. On the day after the final PTH(1‐34) injection, the differentiation status of the osteoblastic microenvironment was evaluated, or basal‐like MDA231 RUNX2‐OE cancer cells were inoculated to evaluate bone colonization. To induce E2‐based various osteoblastic microenvironments, 6‐week‐old female NOD‐SCID mice were administered E2 (2 mg/kg/week; Selleck, S4046) weekly, E2 in combination with dexamethasone (0.5 µg/mL in the drinking water; Selleck, S1322), or E2 and fed a low‐calcium diet (0.01% calcium, 0.4% phosphorus and 2200 IU/kg vitamin D; ENVIGO, TD.95027). After these treatments, the differentiation status of the osteoblastic microenvironment and bone colonization of the mice were evaluated following the inoculation of luminal‐like MCF7 RUNX2‐OE or control cancer cells.

### 4.11 Evaluation of the Differentiation Status of the Bone Microenvironment

To evaluate the differentiation status of the osteoblastic microenvironment, the mice were injected with tetracycline hydrochloride (30 mg/kg; Solarbio, T8180) on days 13 and 14 and with calcein (6 mg/kg; Solarbio, C7600) on days 3 and 4 before sacrifice. After euthanasia, the right hind femurs were scanned via micro‐CT (Faxitron CT system, Hologic, Marlborough, MA, USA), followed by the preparation of nondecalcified histological sections for tetracycline hydrochloride/calcein dual fluorescence detection of the BMR. Using the microCT analysis module, trabecular bone mass was assessed via the bone volume fraction (BV/TV) and trabecular thickness (Tb.Th), whereas the marrow volume (Ma.V) was measured as a reflection of cortical bone mass. Calcein fluorescence represents osteoblast activity, and the distance between yellow tetracycline hydrochloride fluorescence and green calcein fluorescence was used to calculate the bone mineralization rate. Von Kossa staining was employed to visualize mineral deposits, and the staining area (VK. A) was subsequently measured and normalized to the bone surface area. The left hind femurs were decalcified for H&E and Masson's staining. Masson's staining was employed to quantify collagen fibers indicating the osteoid mass via the collagen volume fraction (CVF), which was calculated as the ratio of the positively stained area to the bone surface area.

### 4.12 Bone Colonization of Breast Cancer Cells In Vivo

For human‐derived MDA231 cells, 1×10^5^ luciferase^+^ control cells or RUNX2‐OE cells were intracardially injected into NOD‐SCID mice that had been pre‐induced to develop either an osteogenic premetastatic niche or a gradient osteoblastic microenvironment alongside their respective control mice. For luminal‐like MCF7‐derived cells, E2 (2 mg/kg/week; Selleck, S4046) was administered subcutaneously to support tumor growth. After 1 week of E2 pretreatment, 1 × 10^5^ luciferase^+^ control cells or RUNX2‐OE tumor cells were intracardially injected. To induce spontaneous bone metastasis, 1 × 10^3^ 4T1 control or RUNX2‐OE cells were injected into the mammary fat pads of 6‐week‐old BALB/c mice. On day 21, tumor resection surgery was performed to remove the visible tumor in situ to extend the observation period. Bioluminescence imaging was conducted with a Xenogen IVIS 200 Imaging System (Caliper Life Sciences, Hopkinton, MA, USA) to monitor cell injection and organ colonization.

### 4.13 Reactivation of Bone Marrow DTCs and Micrometastases by the Activation of the Osteoblastic Microenvironment In Vivo

PTH and E2 were used to reactivate DTCs and micrometastases by establishing an active osteoblastic microenvironment. Luciferase^+^ MDA231 RUNX2‐OE cells (1 × 10^5^ cells) were injected into 8‐week‐old female NOD‐SCID mice via intracardiac injection. One week later, when bioluminescence imaging revealed no significant cancer cell colonization and immunohistochemical staining for pan‐CK indicated the presence of DTCs/micrometastases in the bones of the mice, treatment with PTH(1‐34) or E2 was initiated. The mice received daily intraperitoneal injections of 100 µg/kg PTH(1‐34) for 10 consecutive days. Alternatively, two different doses of E2 were administered via subcutaneous injection, including a single low dose of 0.3 mg/kg or three higher doses of 2 mg/kg given weekly. Additionally, ATN‐161 (1 mg/kg, MCE, HY‐13535A) was employed to disrupt the tumor–bone matrix interaction mediated by ITGA5.

### 4.14 Immunohistochemical Staining

Immunohistochemical staining for pan‐CK using formalin‐fixed and paraffin‐embedded tissue sections was conducted to identify DTCs (1–3 tumor cells) or micrometastases (>3 tumor cells) in the bone marrow of mice without osteolytic lesions. A mouse anti‐pan‐CK primary antibody (1:100; RRID: AB_2941997) and a horseradish peroxidase‐conjugated secondary antibody (1:1000; ZSBG‐BIO) were used, with protein visualization achieved via diaminobenzidine.

### 4.15 Multiplexed Fluorescence Immunohistochemical Staining

Paraffin‐embedded tissue sections were deparaffinized and subjected to antigen retrieval. After being blocked with 3% hydrogen peroxide and 10% donkey serum, the sections were incubated overnight at 4 °C with the following primary antibodies: mouse anti‐pan‐CK (1:100; RRID: AB_2941997) and rabbit anti‐KI67 (1:200; RRID: AB_2756525). The sections were then incubated with Alexa Fluor 594‐conjugated anti‐mouse secondary antibodies (1:400; RRID: AB_2534073) and Alexa Fluor 488‐labeled anti‐rabbit secondary antibodies (1:400; RRID: AB_143165) at room temperature for 45 min. After nuclear staining with DAPI (Solarbio, C0060), the cells were observed, and images were captured using a fluorescence microscope (Olympus, Tokyo, Japan).

### 4.16 RNA Sequencing (RNA‐seq)

Total RNA was extracted from cancer cells using TRIzol reagent. Library construction and sequencing were conducted at BGI Genomics (Shenzhen, China). The raw sequencing reads were filtered and aligned to the reference sequences using STAR (v2.4.2a). Three independent RNA extractions and RNA‐seq experiments were performed for each sample. Principal component analysis (PCA) was conducted on the global RNA expression data. Differentially expressed genes (DEGs) between basal‐like MDA231‐derived and luminal‐like MCF7‐derived cells were identified as genes with an average fold change >3.0 and a *p* value ≤0.05. DEGs regulated by RUNX2 overexpression in both MCF7 and MDA231 cells were identified as genes with a fold change >2.0 and a *p* value ≤0.05. DEGs were used for hierarchical clustering and Hallmark gene set and GO biological process enrichment analyses.

### 4.17 Real‐Time RT‐qPCR

Real‐time RT‐qPCR was performed to validate the RNA‐seq results by measuring mRNA expression levels of *ITGA5*, *CDH11*, *TNFSF10* (TNF superfamily member 10), and *NFKB1* (nuclear factor kappa B subunit 1). Expression levels were normalized to the housekeeping gene glyceraldehyde 3‐phosphate dehydrogenase (*GAPDH*). Total RNA was extracted from MDA231‐derived cells and MCF7‐derived cells with TRIzol reagent (Invitrogen, 15596026), and reverse transcription was performed using the cDNA synthesis system (Invitrogen, 18080400). Quantitative PCR was carried out on an ABI 7500 system (Applied Biosystems, Foster City, California) using the SYBR Premix Ex Taq Kit (Takara, DRR041A). Primer sequences were as follows: *ITGA5* forward GCTGGACTGTGGAGAAGAC, *ITGA5* reverse AAGTGAGGTTCAGGGCATTC; *CDH11* forward TCCCTTCACAGCAGAACTAACA, *CDH11* reverse TCACCCACCTCTAAGGCCATC; *TNFSF10* forward TGGCAACTCCGTCAGCTCGTTA, *TNFSF10* reverse AGCTGCTACTCTCTGAGGACCT; *NFKB1* forward GCAGCACTACTTCTTGACCACC, *NFKB1* reverse TCTGCTCCTGAGCATTGACGTC; *GAPDH* forward ATTGTCAGCAATGCATCCTG, *GAPDH* reverse ATGGACTGTGGTCATGAGCC. Relative expression was determined from three independent experiments.

### 4.18 Analysis of Gene Expression Datasets

The outcome and age distributions of bone metastases in breast cancer patients was evaluated using a dataset comprising 295 primary breast cancer tissues from the Netherlands Cancer Institute (NKI295) [56] and another dataset consisting 286 primary breast cancer tissue samples from the Erasmus Medical Center (GSE2034) [57]. Detailed patient information, including the molecular subtypes and occurrence of bone metastasis was gathered from the relevant references [56, 57, 58, 59, 60].

### 4.19 Statistical Analysis

The data are presented as the means ± standard deviations (SDs) from a minimum of three independent experiments. Statistical analyses were conducted using GraphPad Prism 8 software. Chi‐square tests or Fisher's exact tests were used to estimate the differences in the incidence of bone colonization in the mice. All other comparisons were evaluated using analysis of variance (ANOVA) or two‐tailed Student's t‐tests.

## Funding

This work was supported by the National Natural Science Foundation of China (Nos. 82273285 and 81672878) and the Research Program of Tianjin Municipal Health Commission (No. 2021179).

## Conflicts of Interest

The authors declare no conflicts of interest.

## Supporting information




**Supporting File**: advs74269‐sup‐0001‐SuppMat.docx.

**Supporting File**: advs74269‐sup‐0002‐Tables.zip.

## Data Availability

All the data supporting the findings of this study are available within the paper and its Supplementary Material. Further inquiries can be directed to the corresponding author.
