# Supplementary material for: The Osteoblastic Microenvironment Determines the Fate of Breast Cancer Cells Disseminated in the Bone Marrow
Source: Adv Sci (Weinh). 2026 Feb 6;13(22):e09980. doi: 10.1002/advs.202509980 (PMC13088306; doi:10.1002/advs.202509980)
Supplement: Supplementary file 1 — Supporting File: advs74269‐sup‐0001‐SuppMat.docx. [file ADVS-13-e09980-s001.docx]

Supporting Information

The Osteoblastic Microenvironment Determines the Fate of Breast Cancer Cells Disseminated in the Bone Marrow

Hong-Li Wang, Rui Zhang, Xiao-Min Yue, Jie Zhou, Yu-Fan Huang, Rong Meng, Yu-Li Wang, Xiao-Qing Li*

Email: xqli@tmu.edu.cn


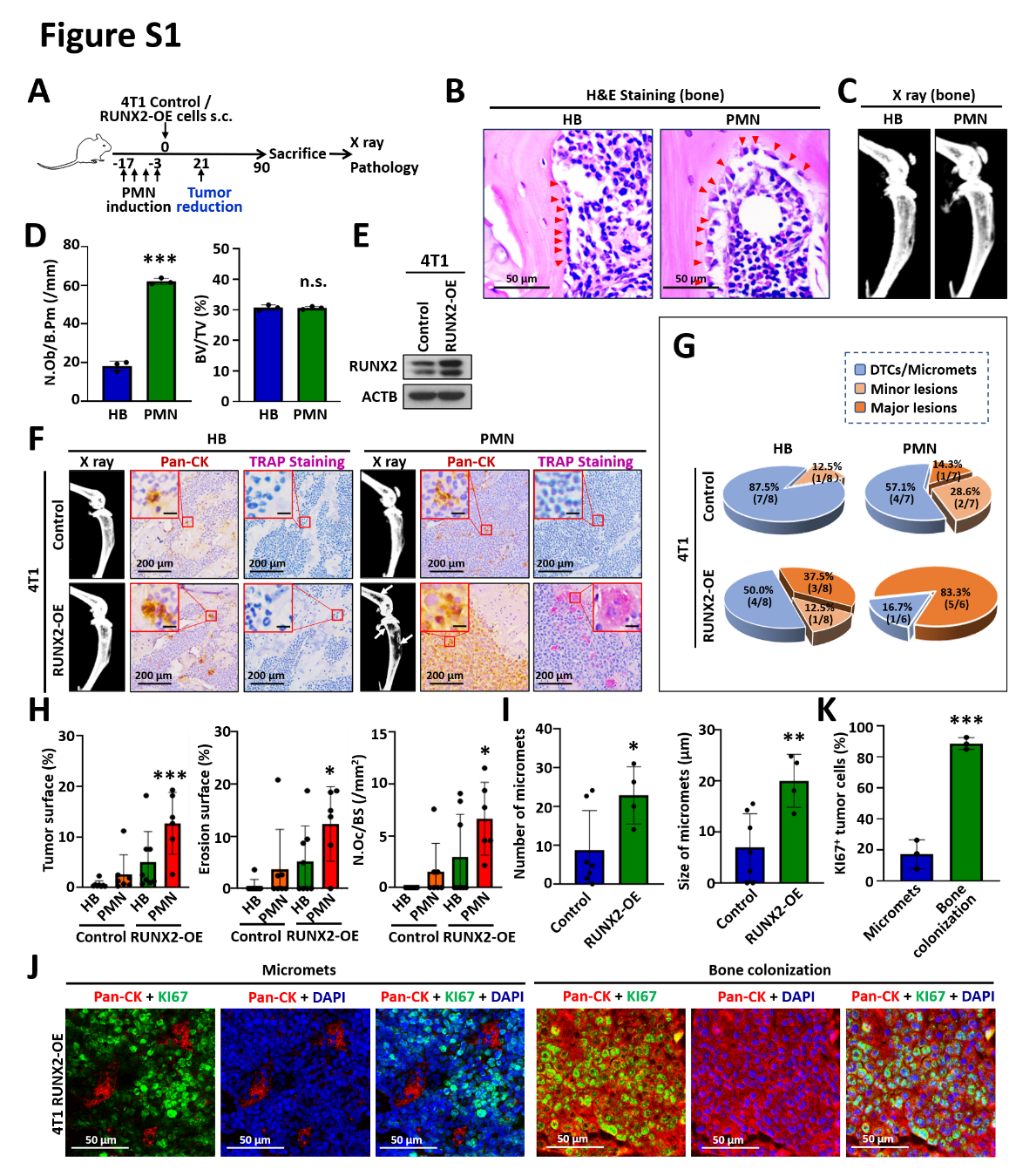


**Figure S1** **RUNX2 facilitates the residency of basal-like 4T1 cells as DTCs, micrometastases, or small osteolytic metastases in the bone marrow of adult mice. A**, Experimental schedule for bone colonization by 4T1-derived cells in BALB/c mice. An osteogenic premetastatic niche (PMN) in BALB/c mice was established by injecting 4T1 CDH11^high^/ITGA5^high^ extracellular vesicles *via* the tail vein for 2 weeks (10 µg/dose, 2 doses/week). 4T1 RUNX2-OE cells and control cells were administered to mice *via* subcutaneous injection into the fat pad. Tumor resection surgery was performed on day 21 to remove the visible tumor *in situ*, with the aim of prolonging the observation period. **B**, H&E staining images demonstrating osteoblasts (indicated by red triangles) in homeostatic bone (HB) and the PMN. **C**, X ray images showing the bone mass in the microenvironment of HB and PMN. **D**, Bar charts quantifying the osteoblast number as the ratio of osteoblast count to bone perimeter in /mm (N.Ob/B.Pm) and bone mass as the bone volume fraction (BV/TV). **E**, Western blot analysis of RUNX2 protein expression in 4T1 RUNX2-OE and control cells. **F**, Representative X ray, pan-CK immunohistochemical staining, and TRAP staining images demonstrating the sizes of the bone metastasis lesions, tumor cell distributions, and numbers of activated osteoclasts, respectively. The scale bars in the inset images indicate 20 µm. **G**, Pie charts depicting the incidences of DTCs, micrometastases (micromets) and osteolytic lesions formed by 4T1 control cells and RUNX2-OE cells within the HB and the PMN. **H**, Bar charts quantifying the tumor surface, erosion surface and the number of TRAP^+^ osteoclasts normalized to the total bone surface (N.Oc/BS in /mm^2^). **I**, Bar charts illustrating the abundance and size of micrometastases in the bone marrow of mice without detectable bone lesions. **J**, Representative KI67 immunofluorescence images demonstrating the slow proliferation of micrometastases and rapid proliferation of cancer cells within bone lesions. Pan-CK was used to label the tumor cells, and DAPI was used to stain the nuclei. **K**, Bar chart illustrating the reduced presence of KI67^+^ tumor cells in micrometastases compared with tumor cells within bone lesions.

The data are displayed as the means ± SDs. **P* < 0.05, ***P* < 0.01 and ****P* < 0.001 compared with the control group, as determined by Student’s t test.


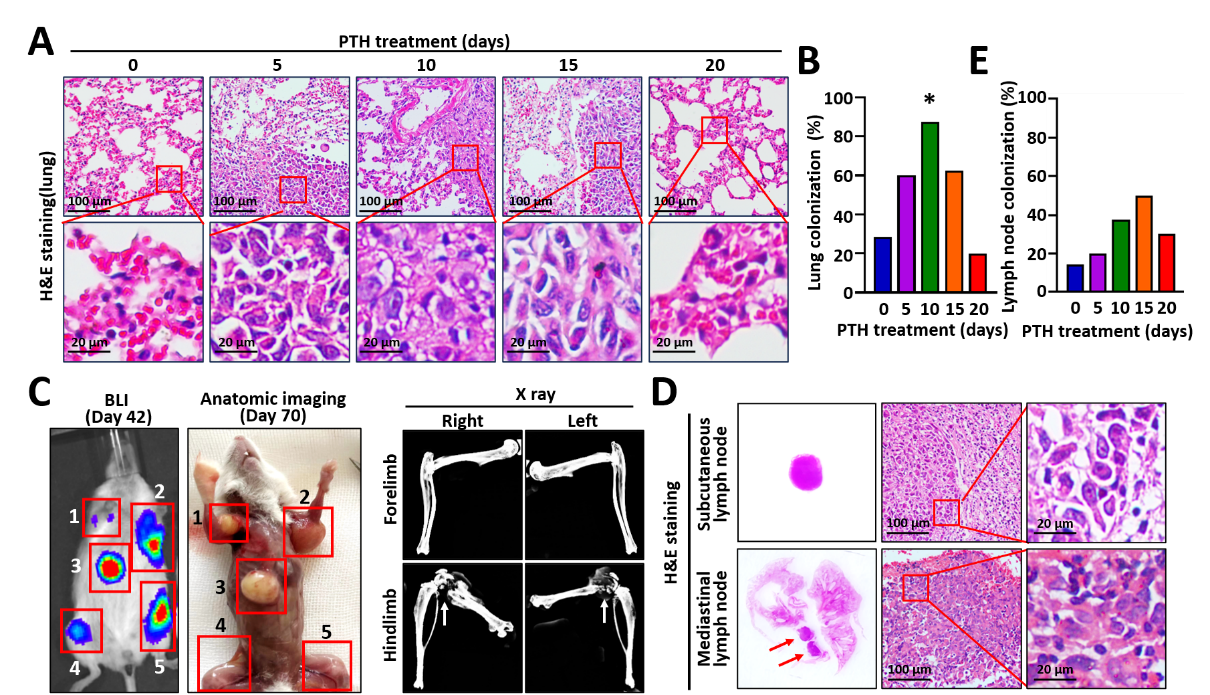


**Figure S2 PTH treatment for 10 days facilitates the lung and lymph node colonization of MDA231 RUNX2-OE cells. A**, Representative H&E staining images showing pulmonary colonization in mice treated with PTH. **B**, Bar chart displaying the incidence of lung colonization in mice treated with PTH for 0, 5, 10, 15 and 20 days. **P*<0.05 was determined using Fisher's exact probability method. **C**, Representative bioluminescence (BLI), autopsy, and X ray images showing the lymph nodes and bone lesions in the same mouse. Loci 1, 2, and 3 indicate lymph node colonization without involvement of the right or left forelimbs, whereas loci 4 and 5 indicate bone colonization without accompanying lymph node swelling. **D**, Representative H&E staining images showing cancer cell colonization in the subcutaneous and mediastinal lymph nodes. Red arrows indicate mediastinal lymph nodes. **E**, Bar chart showing the incidence of lymph node colonization in mice treated with PTH.


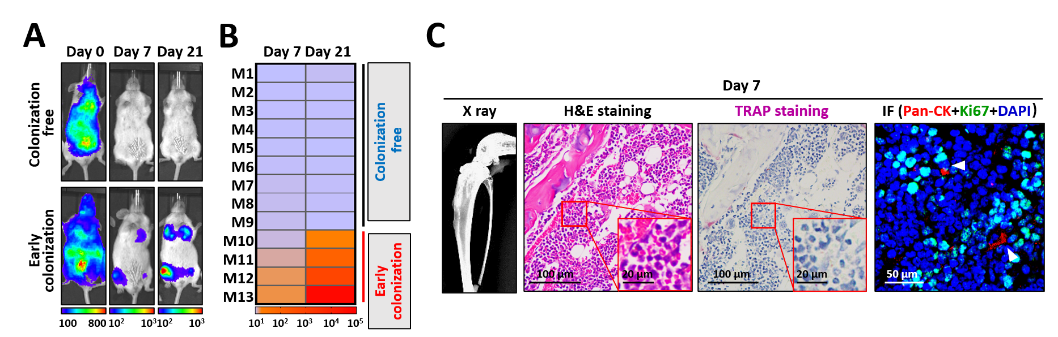


**Figure S3 MDA231 RUNX2-OE cells persist as DTCs or micrometastases in the bone marrow of mice 7 days after intracardiac injection.** **A**, Representative BLI images illustrating the systemic distribution on day 0 and subsequent tumor cell persistence in mice following intracardiac inoculation on days 7 and 21. **B**, Heatmap depicting the BLI intensity in mice on days 7 and 21 following tumor cell injection. **C**, X ray, H&E staining, TRAP staining, and pan-CK and KI67 multiplexed fluorescence immunohistochemical staining images showing the presence of quiescent DTCs/micrometastases without accompanying bone lesions or osteoclast activation on day 7. The white triangles indicate CK^+^ tumor cells.


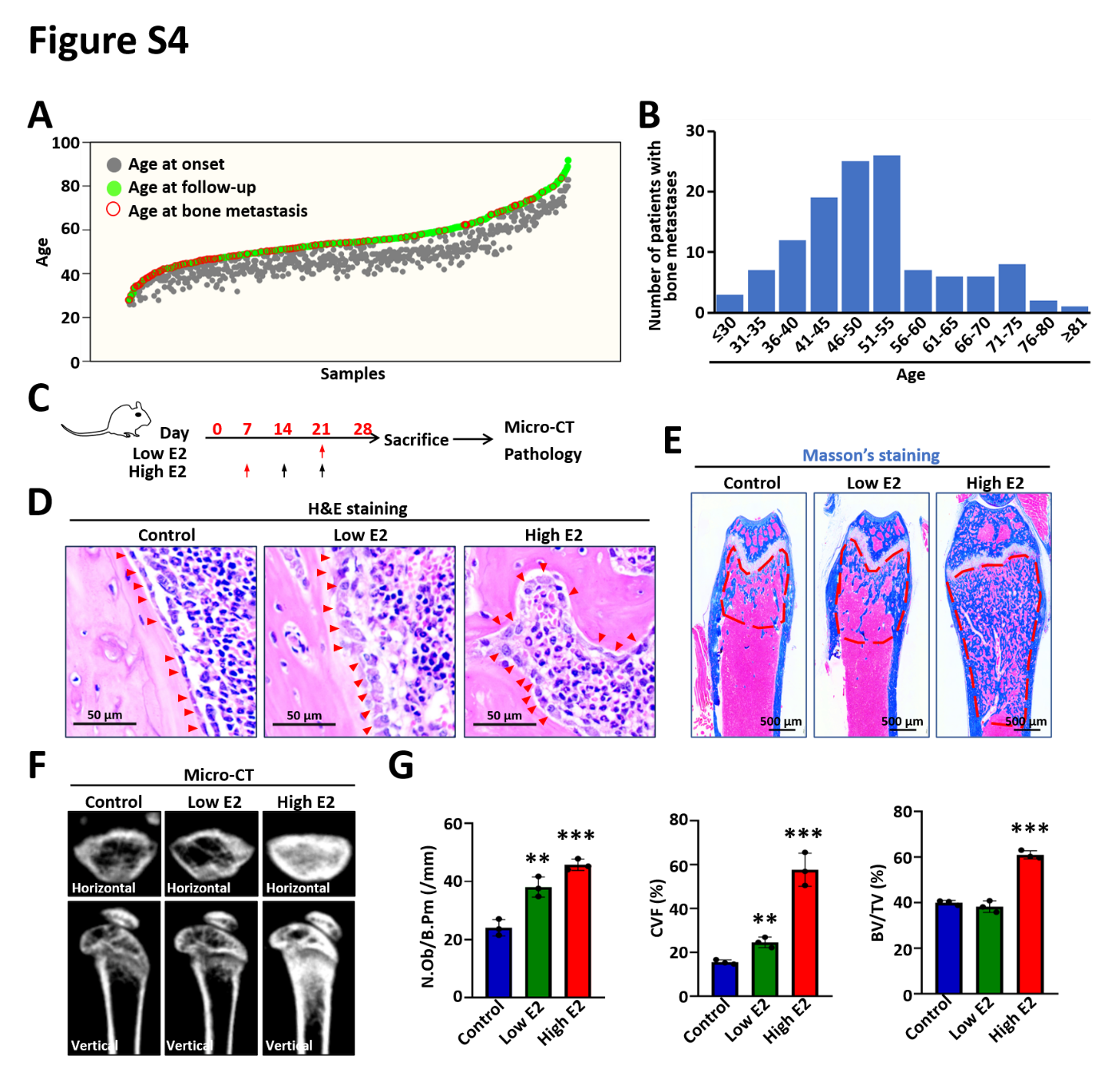


**Figure S4 Age distribution at the occurrence of bone metastasis and effects of estradiol cypionate (E2) on osteoid accumulation and bone mass. A**, Scatterplot showing the age at onset (gray dots), age at follow-up (green dots), and age at bone metastasis (red circles) for breast cancer patients in the GSE2034 and NKI295 datasets. **B**, Histogram showing the age distribution of breast cancer patients at the time of bone metastasis in the GSE2034 and NKI295 datasets. **C**, Experimental schedule for E2-induced activation of the osteoblastic microenvironment. Two different doses of E2 were administered via subcutaneous injection. High-dose E2 (2 mg/kg) was given weekly for 3 weeks, while a single low-dose E2 (0.3 mg/kg) was administered on day 21, concurrently with the final dose in the high-dose group. Mice injected with an equal volume of the solvent corn oil served as controls. One week later, the mice were sacrificed for micro-CT imaging and histological staining analyses. **D-G**, Representative H&E staining (**D**), Masson’s staining (**E**), and micro-CT (**F**) images and bar charts (**G**) indicating the number of osteoblasts (N.Ob/B.Pm), collagen volume fraction (CVF), and bone volume fraction (BV/TV) in mice receiving either low-dose E2 or high-dose E2, along with control mice.

The data are displayed as the means ± S0Ds. ***P* < 0.01 and ****P* < 0.001 compared with the control group, as determined by Student’s t test.


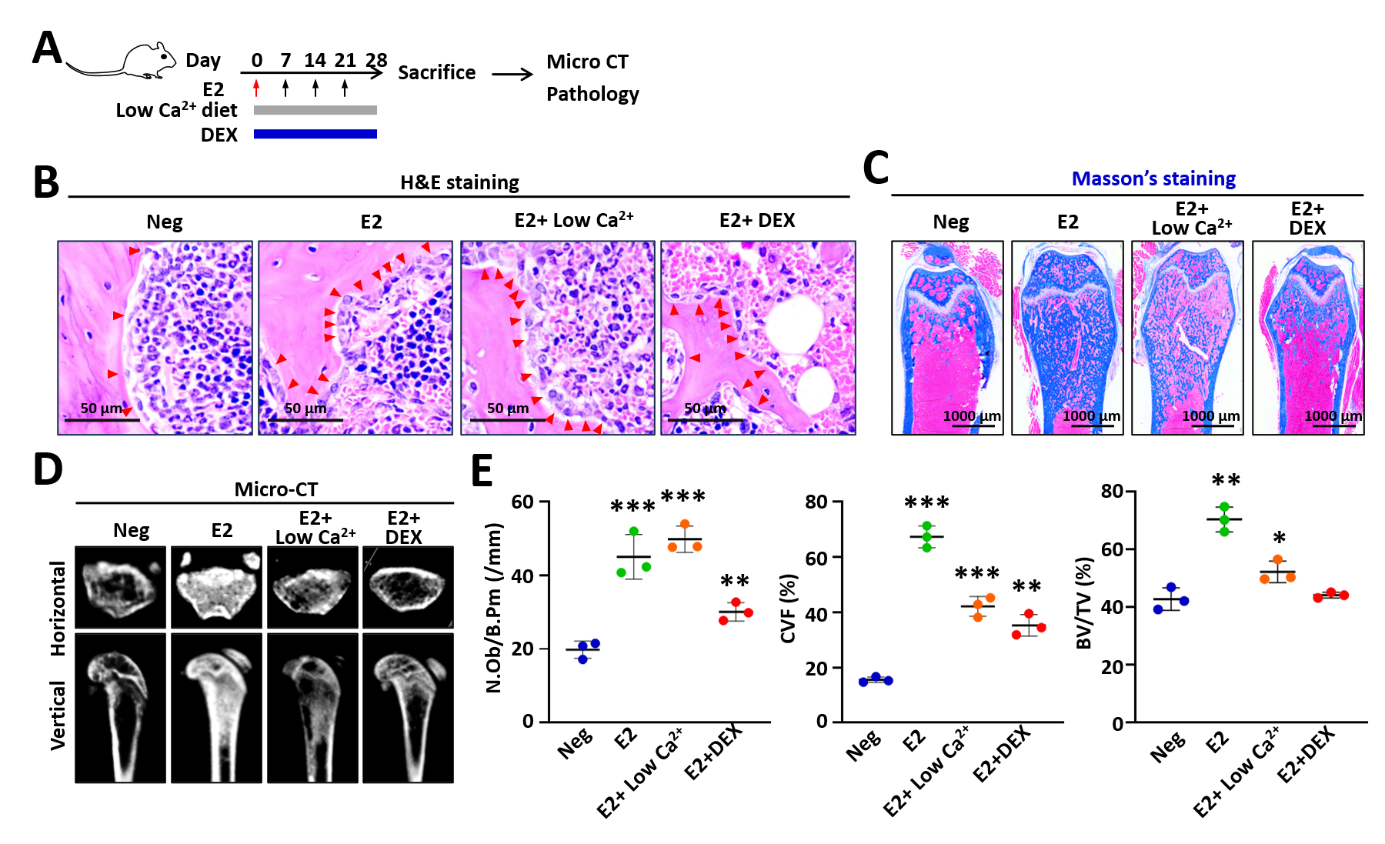


**Figure S5 Induction of E2-based various osteoblastic microenvironments. A**, Experimental diagram illustrating the induction of various osteogenic microenvironments. SCID mice were administered E2 (2 mg/kg) weekly, either alone or in combination with dexamethasone (0.5 µg/mL in drinking water) or feeding a low-calcium diet for 4 weeks. The differentiation status of the osteoblastic microenvironment was then evaluated *via* micro-CT and Masson’s staining. **B**, Representative H&E staining images demonstrating osteoblasts (indicated by red triangles). **C**, Representative images of Masson’s staining. **D**, Representative images of micro-CT scans. **E**, Strip charts showing the osteoblast number as N.Ob/B.Pm, the CVF quantified by Masson’s staining and the BV/TV determined from micro-CT images.


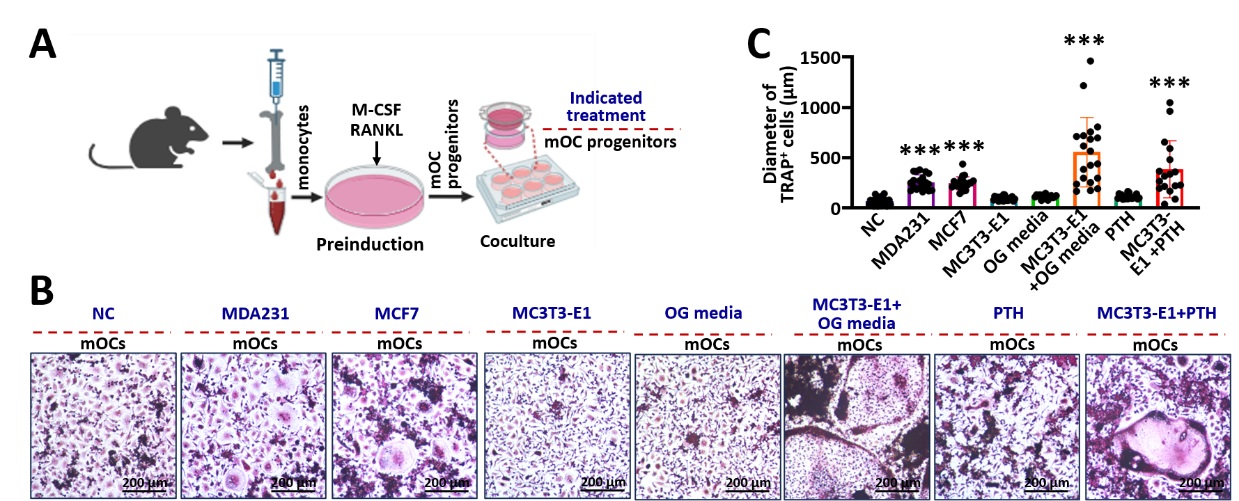


**Figure S6 Activation of osteoclasts by tumor cells and well differentiated osteoblasts. A**, Schematic representation of the two-chamber *in vitro* coculture system. Mouse primary osteoclast (mOC) progenitors were generated by inducing primary mouse monocytes with 50 ng/mL M-CSF for 2 days. The mOC progenitors were then seeded into the lower chamber together with 50 ng/mL RANKL. Moreover, the indicated cells, osteogenic media (OG media), or PTH was added to the upper chamber for an additional 5 days of incubation. The activity of the mOCs was evaluated *via* TRAP staining. **B-C**, Representative TRAP staining images and a bar chart showing the osteoclast activity of mouse primary osteoclasts (mOCs) cocultured with tumor cells and MC3T3-E1 osteoblasts. Well differentiated MC3T3-E1 osteoblasts were obtained by inducing MC3T3-E1 cells in osteogenic medium (OG medium supplemented with 50 μg/mL L-ascorbic acid and 10 mM β-glycerophosphate) or with 0.5 ng/mL PTH. The data are displayed as the means ± SDs. ****P* < 0.001 compared with the negative control (NC), as determined by Student’s t test.


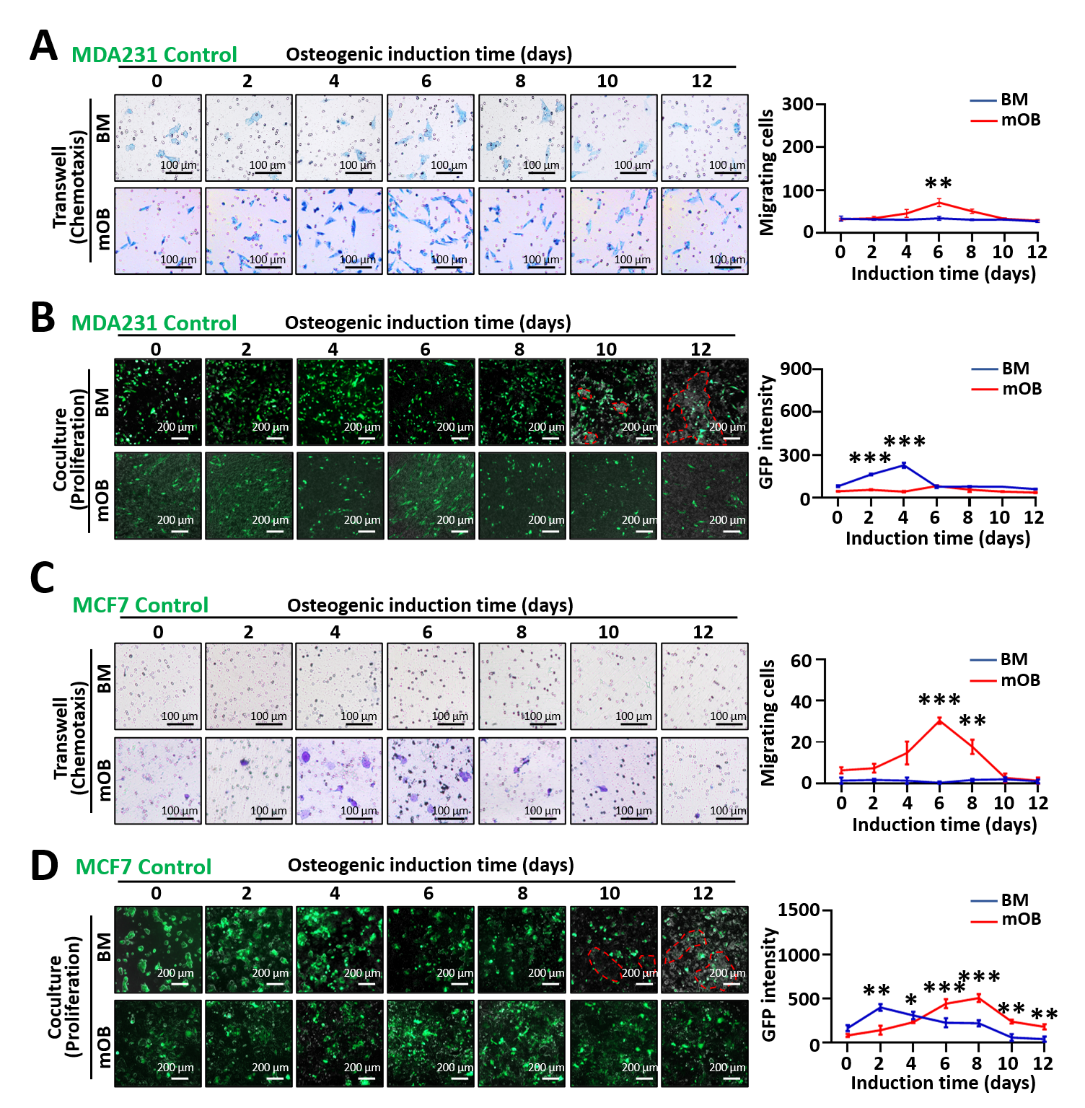


**Figure S7 Osteoblasts and the bone matrix exhibit distinct effects on control breast cancer cells *in vitro* in the osteoblastic microenviroment**. **A-B**, Effects of mouse primary osteoblasts (mOBs) and the bone matrix (BM) at different stages of differentiation on the chemotactic (**A**) and proliferative (**B**) capacities of GFP-labeled MDA231 control cells. Calcium nodules are circled in red dashed lines. **C-D**, Effects of mOBs and the BM at different stages of differentiation on the chemotactic (**C**) and proliferative (**D**) capacities of GFP-labeled MCF7 control cells. Calcium nodules are circled with red dashed lines.

The data are presented as the means ± SDs. **P* < 0.05, ***P* < 0.01, ****P* < 0.001 compared with the respective control group (0 days), as determined by Student’s t test.


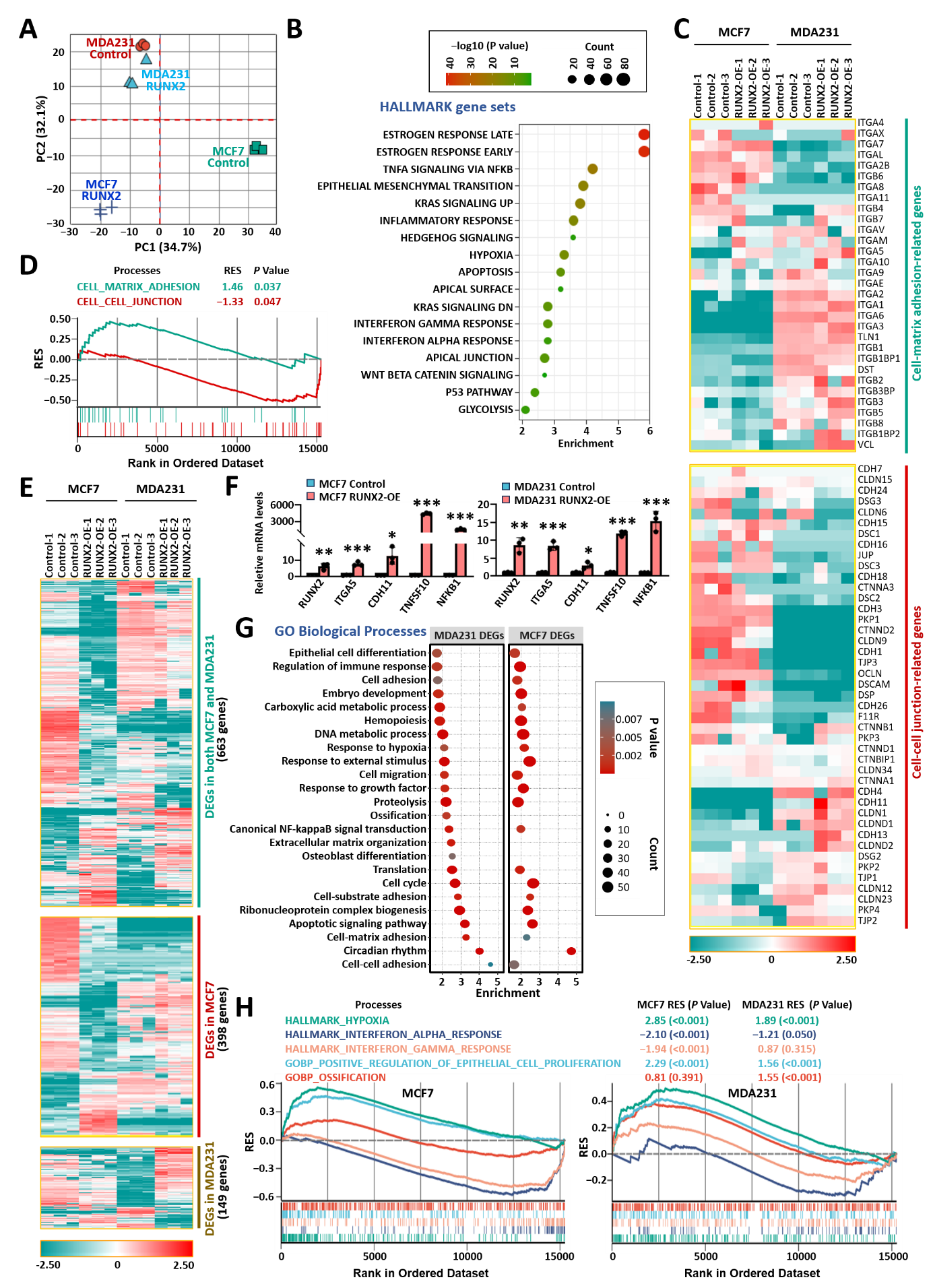


**Figure S8 Differences in gene expression profiles between MDA231 and MCF7 cells and the molecular changes regulated by the overexpression of RUNX2.** The transcriptional profiles of RUNX2-overexpressing MDA231 and MCF7 cells, as well as their respective control cells, were investigated using bulk RNA sequencing. **A**, Principal component analysis (PCA) revealing differences in the transcription profile between MDA231-derived cells and MCF7-derived cells. **B**, Enrichment of differentially expressed genes (DEGs) between MDA231-derived and MCF7-derived cells in HALLMARK gene sets. **C**, Heatmap displaying the enrichment of cell–matrix adhesion-related and cell–cell junction-related genes in MCF7-derived and MDA231-derived cells. **D**, Gene set enrichment analysis (GSEA) showing the enrichment of the DEGs in cell–matrix adhesion and cell–cell junction gene sets between MDA231-derived cells and MCF7-derived cells. **E**, Heatmap illustrating the changes in the expression levels of mRNA regulated by RUNX2 overexpression, including DEGs in both MCF7 and MDA231, as well as DEGs specific to each cell line. **F**, RT-qPCR validating the mRNA expression level of RUNX2-regulated genes *ITGA5*, *CDH11*, *TNFSF10* and *NFKB1*. **G**, GO biological process analysis of DEGs regulated by RUNX2 overexpression in MCF7 and MDA231 cells. **H**, GSEA of the DEGs between MCF7 RUNX2-OE and MDA231 RUNX2-OE cells compared with their respective control cells.
